# Japan Society of Gynecologic Oncology guidelines 2015 for the treatment of vulvar cancer and vaginal cancer

**DOI:** 10.1007/s10147-017-1193-z

**Published:** 2017-11-20

**Authors:** Toshiaki Saito, Tsutomu Tabata, Hitoshi Ikushima, Hiroyuki Yanai, Hironori Tashiro, Hitoshi Niikura, Takeo Minaguchi, Toshinari Muramatsu, Tsukasa Baba, Wataru Yamagami, Kazuya Ariyoshi, Kimio Ushijima, Mikio Mikami, Satoru Nagase, Masanori Kaneuchi, Nobuo Yaegashi, Yasuhiro Udagawa, Hidetaka Katabuchi

**Affiliations:** 1 Gynecology Service, National Kyushu Cancer Center, Fukuoka, Japan; 2 Department of Obstetrics and Gynecology, Graduate School of Medicine, Mie University, Mie, Japan; 3 Department of Therapeutic Radiology, Tokushima University, Tokushima, Japan; 4 Department of Diagnostic Pathology, Okayama University Hospital, Okayama, Japan; 5 Department of Obstetrics and Gynecology, Faculty of Life Sciences, Kumamoto University, Kumamoto, Japan; 6 Department of Obstetrics and Gynecology, Tohoku University Graduate School of Medicine, Sendai, Japan; 7 Department of Obstetrics and Gynecology, Graduate School of Comprehensive Human Sciences, University of Tsukuba, Tsukuba, Japan; 8 Department of Obstetrics and Gynecology, Tokai University School of Medicine, Kanagawa, Japan; 9 Department of Gynecology and Obstetrics, Kyoto University Graduate School of Medicine, Kyoto, Japan; 10 Department of Obstetrics and Gynecology, Keio University School of Medicine, Tokyo, Japan; 11 Department of Obstetrics and Gynecology, Kurume University School of Medicine, Kurume, Japan; 12 Department of Obstetrics and Gynecology, Faculty of Medicine, Yamagata University, Yamagata, Japan; 13 Department of Obstetrics and Gynecology, Nagasaki University Graduate School of Medicine, Nagasaki, Japan; 14 Department of Obstetrics and Gynecology, Fujita Health University School of Medicine, Aichi, Japan

**Keywords:** Clinical practice guidelines, Vulvar cancer, Vaginal cancer, Vulvar Paget’s disease, Malignant melanoma of the vulva and vagina, Treatment

## Abstract

**Background:**

Vulvar cancer and vaginal cancer are relatively rare tumors, and there had been no established treatment principles or guidelines to treat these rare tumors in Japan. The first version of the Japan Society of Gynecologic Oncology (JSGO) guidelines for the treatment of vulvar cancer and vaginal cancer was published in 2015 in Japanese.

**Objective:**

The JSGO committee decided to publish the English version of the JSGO guidelines worldwide, and hope it will be a useful guide to physicians in a similar situation as in Japan.

**Methods:**

The guideline was created according to the basic principles in creating the guidelines of JSGO.

**Results:**

The guidelines consist of five chapters and five algorithms. Prior to the first chapter, basic items are described including staging classification and history, classification of histology, and definition of the methods of surgery, radiation, and chemotherapy to give the reader a better understanding of the contents of the guidelines for these rare tumors. The first chapter gives an overview of the guidelines, including the basic policy of the guidelines. The second chapter discusses vulvar cancer, the third chapter discusses vaginal cancer, and the fourth chapter discusses vulvar Paget’s disease and malignant melanoma. Each chapter includes clinical questions, recommendations, backgrounds, objectives, explanations, and references. The fifth chapter provides supplemental data for the drugs that are mentioned in the explanation of clinical questions.

**Conclusion:**

Overall, the objective of these guidelines is to clearly delineate the standard of care for vulvar and vaginal cancer with the goal of ensuring a high standard of care for all women diagnosed with these rare diseases.

**Electronic supplementary material:**

The online version of this article (doi:10.1007/s10147-017-1193-z) contains supplementary material, which is available to authorized users.

## Introduction

Although vulvar cancer and vaginal cancer are rare tumors, they actually constitute the fourth and fifth most common cancers in the gynecologic oncology field. However, there are no established treatment principles or guidelines to treat these rare tumors in Japan. The Guideline Committee of Japan Society of Gynecologic Oncology (JSGO) has established the treatment guidelines for cervical cancer and considered further revisions to the Guidelines for the Treatment of Cervical Cancer. However, as the revised version would include new content about treatment guidelines for vulvar and vaginal cancer that had not been made explicit internationally in 2015, it was decided to publish a fourth series of JSGO treatment guidelines, the Guidelines for the Treatment of Vulvar and Vaginal Cancer, 2015 edition. In the course of preparing the guidelines, a period just under 2 years, the Committee searched extensively for data on the treatment of vulvar and vaginal cancer, both in Japan and internationally, and collated the findings. Finally, treatment guidelines that the committee felt were most applicable to treatment situations in Japan were presented in the form of 16 clinical questions. The committee is confident that the guidelines constitute an indispensable resource for healthcare providers engaged in the treatment of vulvar and vaginal cancer, and that their use will lead to the best possible outcomes for cancer patients and their families.

The main points of these treatment guidelines are as follows.

1. Clinical questions (CQs) are established with focus on vulvar and vaginal cancer, which are epithelial neoplasias. Rare malignancies that can be treated by gynecologic oncologists, such as vulvar Paget’s disease, as well as malignant melanomas are also included as objects of consideration. To encompass all of these types of cancers, the broad terms ‘vulvar cancer’ and ‘vaginal cancer’ were adopted.

2. The ‘Basic Items Regarding The Guidelines' section provides explanations regarding staging classification, histological categories, methods of surgical therapies, radiation therapies and chemotherapies, to give the reader a better understanding of the contents of the guidelines (supplied as an Appendix in the present article).

3. The ‘Basic Items’ section provides historical background on the classification of staging in vulvar and vaginal cancer. It lists the classifications of staging adopted by the JSOG in 2014 and sets in order the names of lymph nodes and their definitions. In addition, in view of the fact that no unique classifications of staging have been developed for vulvar malignant melanomas, the TNM classifications for cutaneous malignant melanomas are adopted with modifications.

4. In histological classifications, because no classifications unique to Japan exist, the original texts are provided of the long-used 2003 World Health Organization (WHO) classifications and the new, revised classifications of 2014. An explanation is added regarding the differences between these two classification systems as regards intraepithelial neoplasias of the vulva and vagina.

5. In surgical therapy and radiation therapy, the terminology in Japanese and English are presented side-by-side. For surgical therapy, the terminology for generally used excision margins and surgical resection stumps is specified.

Following publication of the Japanese guidelines in August 2015, the NCCN Clinical Practice Guidelines in Oncology published the guidelines for vulvar cancer (squamous cell carcinoma) version 1.0 on the website in January 2016. Although the basic principles were almost identical to the JSGO guidelines, differences existed in several points. The major difference is that the JSGO guidelines include vaginal cancer and other diseases in the vulva and vagina, such as Paget’s disease and malignant melanoma, and intend to publish for gynecologists who are not familiar with these rare diseases in Japan. The committee decided to publish the English version of the JSGO guidelines worldwide, and hope it will be a useful guide to physicians in a similar situation in Japan.

## Chapter 1: Overview of guidelines

1. How to use these guidelines

The purpose of the guidelines is to indicate one set of standards that can be used to select better options in the treatment of vulvar and vaginal cancer in Japan, and to provide the evidence on which those options are based. However, the guidelines are not intended to limit the therapies listed. The principal objectives of the guidelines are (1) to indicate treatment methods that are currently considered appropriate for vulvar and vaginal cancer; (2) to minimize variances in the treatment methods among institutions; (3) to improve the safety of treatment and prognosis of the diseases; (4) to reduce the economic and psychosomatic burden of patients by promoting the performance of appropriate treatment; and (5) to enhance mutual understanding between patients and healthcare professionals.

2. Intended readers

The guidelines are for the use of all physicians involved in the treatment of vulvar and vaginal cancer.

3. Diseases addressed by these guidelines

In the guidelines, the term ‘vulvar and vaginal cancer’ covers not only epithelial tumors such as vulvar cancer, vaginal cancer, but also vulvar Paget’s disease and malignant melanomas of the vulva and vagina.

4. Basic principles in creating the guidelines

To prepare the guidelines, two independent committees, the Committee for Drafting of the Guidelines for the Treatment of Vulvar and Vaginal Cancer and the Committee for Evaluation of the Guidelines for the Treatment of Vulvar and Vaginal Cancer (‘Drafting Committee’ and ‘Evaluation Committee’) were established within the Guideline Committee established by the JSGO. The first draft of the guidelines was created as a result of extensive research by both of these committees. Thereafter, the opinions of the Japan Society of Obstetrics and Gynecology (JSOG), the Japan Association of Obstetrics and Gynecology (JAOG), the Japanese Gynecologic Oncology Group (JGOG), the Japanese Society for Radiation Oncology (JASTRO), the Japanese Society of Pathology (JSP), the Japanese Dermatological Association (JDA), the Japan Society of Plastic and Reconstructive Surgery (JSPRS), the Japan Society of Clinical Oncology (JSCO) and other related academic societies and associations were incorporated into the document to produce a final draft. After the final draft was circulated among JSGO members and a consensus was reached, the JSGO approved the draft for publication.

Most of the evidence adopted in the guidelines was obtained from clinical trials conducted in North America, Europe and Japan. However, given the differences between practices in Japan and other countries, the consensus regarding clinical practice in Japan took priority in the event of discrepancies.

In addition, the following general principles guided the committee’s drafting policy.

1. The guidelines were prepared in accordance with the procedures of evidence-based medicine, the international standard method.
2. The committee gathered and accumulated evidence through extensive perusal of literature and data reported throughout Japan and worldwide up to December 2013. Where deemed necessary, evidence was also adopted from literature and data reported during the drafting period of the guidelines, subsequent to December 2013.
3. The collected evidence was evaluated for quality using the criteria of the JSCO and its Formulation Committee of Clinical Practice Guidelines for the Use of Anticancer Agents [1, 2]. However, some of the contents were modified in line with these guidelines (Table 1).

**Table 1:** Criteria for the evaluation of the quality of evidence (levels)

Level I	Meta-analysis of multiple randomized controlled trials
Level II	Randomized controlled trials or well-designed non-randomized controlled trials
Level III	Well-designed quasi-experimental studies, comparative studies, correlation studies, case-comparison studies or other well-designed non-experimental descriptive studies
Level IV	Reports and opinions of specialized committees or clinical experiences of authoritative persons
4. The criteria of the strength for the recommendations indicated in the guidelines are determined by the criteria for recommendations in JSCO’s Guidelines for the Appropriate Use of Anti-cancer Drugs [1, 2] and the Minds Handbook on Preparation of Treatment Guidelines 2007 [3]. However, some of the content of those guidelines have been modified for the purposes of the guidelines (Table 2).

**Table 2:** Criteria for recommendation (grades)

Grade A	Action is strongly recommended In principle at least one Level I item of evidence indicating effectiveness is present
Grade B	Action is recommended In principle at least one Level II item of evidence indicating effectiveness is present
Grade C1	Action may be considered, but scientific grounds are not yet sufficient (Alternatively, scientific grounds are not yet sufficient, but the possibility exists that effectiveness can be expected) Multiple Level III items of evidence indicating effectiveness are present, and results are generally consistent
Grade C2	Scientific grounds are not sufficient and application in routine treatment is not recommended
Grade D	Action is not recommended Usefulness/effectiveness is not evident, and indeed the treatment may be harmful

In addition to the question of evidence, recommendation Grade A can be applied based on judgment on the level of general common sense. Because evidence is extremely sparse in the case of rare diseases, recommendation grades are decided based on the judgment of the Drafting Committee
5. Each of the topics in the guidelines consists of a CQ, a recommendation, an objective or set of objectives, and an explanation. If a more detailed explanation is deemed necessary to recommend a treatment, the explanation is added in a supplementary note.
6. At the end of each topic, the literatures which the guideline cited were collated in Literature Cited.
7. Some of the treatment methods evaluated and recommended worldwide are problematic in terms of application under Japan’s medical insurance system. In this regard, the present guidelines follow the Formulation Committee of Clinical Practice Guidelines for the Use of Anticancer Agents [1, 2].

5. Disclosure of information

The guidelines are intended to be used widely. Their content is published as a booklet and is available for perusal on the JSGO website.

6. Responsibility for treatment

The JSGO bears the responsibility for the content and description of these guidelines. However, the final decision to use these guidelines should be made by the individual user. Thus, the responsibility for the treatment outcomes should be directly attributed to the person in charge.

7. Revision

1. These guidelines are continuously being revised by the Committee for Treatment Guidelines for Vulvar and Vaginal Cancer with medical advances and medical changes.
2. Newly accumulated evidence after publishing these guidelines is saved in a database.
3. Any associated information regarding clinical inconvenience occurring with the use of these guidelines is collected.
4. Revision work is conducted by the Guidelines Formulation Committee and Evaluation Committee based on new evidence and information. Moreover, opinions from the associated academic societies, groups or JSGO members are widely gained.
5. After the processes described above, the Drafting Committee organizes the final revisions to the guidelines, and the JSGO approves the draft.

8. Summary of recommendation

In general, each chapter comprises a CQ, recommendations, background, objectives, explanations, and references. This article summarizes these guidelines in a question-and-answer format. Recommendations from each chapter are listed below under their respective chapter titles.

9. Algorithms

These guidelines contain the following 5 algorithms:

1. Primary treatment for vulvar cancer (vulvar tumor and inguinal lymph node)
2. Management of inguinal lymph nodes of vulvar cancer
3. Treatment of distant metastasis, recurrent tumor of vulvar cancer
4. Primary treatment for vaginal cancer
5. Primary vulvar Paget’s disease

10. Nomenclatures and classifications

1. Staging of the disease, FIGO staging and TNM staging
2. WHO histopathological classification (2014)
3. Nomenclature and definition of surgical treatment and methods
4. Nomenclature and definition of radiation therapy

## Chapter 2: Treatment strategies for vulvar cancer

### General consideration

The curative treatment of vulvar cancer requires consideration of both the primary focus of disease in the vulva and the inguinal lymph nodes, which are the regional lymph nodes of the vulva. In current treatment, surgery is the first choice, and historically there has been a transition away from radiation therapy toward surgical procedures in Japan [4, 5]. The FIGO classifications of stages also consist of classifications of surgical staging, which include detailed histopathological findings on lymph node metastasis. However, radiation therapy remains a frequently chosen option, as patients with vulvar cancer are typically elderly and have a high rate of medical complications and high risk for surgery. For cases with high-risk factors for postoperative recurrence, postoperative adjuvant radiation therapy is performed. As with uterine cancer, concurrent chemoradiotherapy (CCRT) is sometimes tried, and combination chemotherapy may be conducted in advanced cases and cases of recurrence (CQ08, CQ10).

**Fig. 1:**
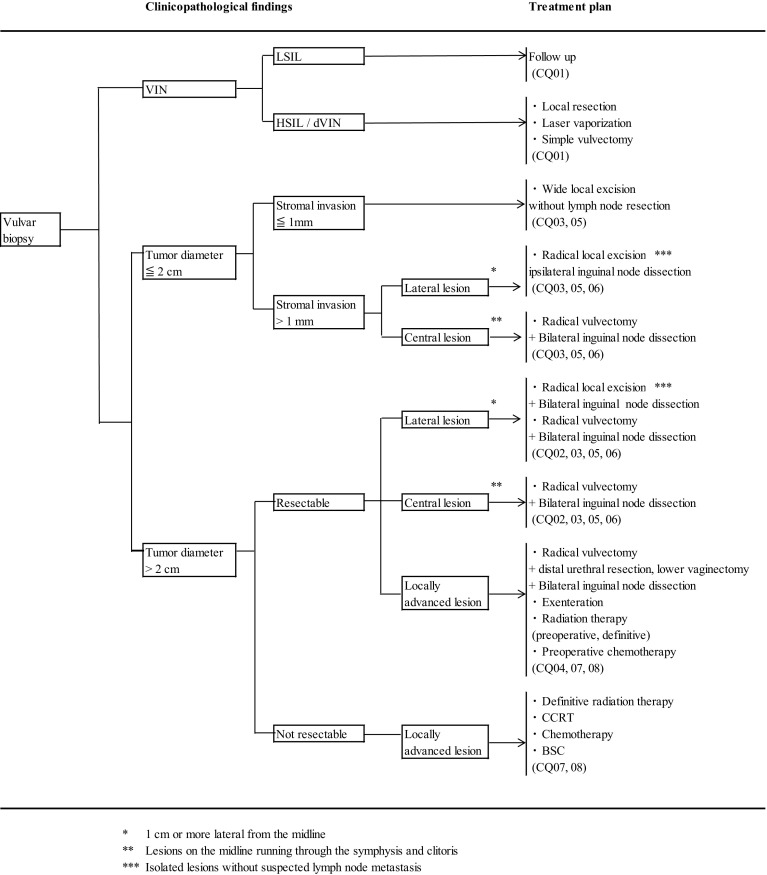
Primary treatment for vulvar cancer (vulvar tumor and inguinal lymph node)

In recent years, efforts have been paid for individualization and reduction of radical treatment with special emphasis on the post-treatment quality-of-life (QOL) of the patients. However, these demands have sometimes led to confusion for the physicians treating vulvar cancer and consistent guidelines for determining the treatment plans are needed (Fig. 1)

(1) Histopathological approaches

Most epithelial tumors of the vulva are derived from squamous epithelium. These are divided into noninvasive vulvar intraepithelial neoplasias (VINs) and squamous cell carcinomas. Human papillomavirus (HPV) is detected in approximately 52–100% of cases of VIN [6]. Formerly, VIN was classified into three categories (VIN 1, VIN 2 and VIN 3) based on the degree of spread of atypical cells. However, in the 2004 classifications of the International Society for the Study of Vulvovaginal Disease (ISSVD), VIN was divided into ‘usual VIN (uVIN), VIN caused by infection with HPV, ‘differentiated VIN (dVIN), and VIN not caused by HPV infection [7]. In the WHO classification (4th edition) published in 2014, VIN related to HPV is called ‘squamous intraepithelial lesion’ (SIL). SIL is itself divided into two types—low-grade SIL (LSIL), thought to be non-neoplastic morphological abnormalities associated with HPV infection, and high-grade SIL (HSIL), neoplastic lesion which carries the risk of carcinogenesis. LSIL corresponds to (HPV-related) VIN 1, and HSIL corresponds to VIN 2 and 3 [8]. Bowenoid papulosis (BP) caused by HPV infection is a black pimple or papule that breaks out repeatedly on the vulva. Although the histological appearance of BP is identical to that of uVIN or HSIL, it disappears spontaneously, so the term BP is not used in histological diagnosis. All these lesions have long attracted attention for their potential to undergo malignant change, and the diagnosis and management of VIN is a matter of importance (CQ01).

Squamous cell carcinoma is divided into five types—keratinizing, non-keratinizing, basaloid, warty and verrucous. The vulvar squamous cell carcinoma seen in young women is frequently caused by infection with HPV and often presents in basaloid or warty form. By contrast, vulvar squamous cell carcinoma in elderly women is rarely caused by HPV infection and is usually keratinizing or non-keratinizing [9].

(2) Surgical treatment

Historically, the 5-year survival rate for treatment of vulvar cancer improved to 60–70% as a result of the establishment of radical vulvectomy and systemic lymphadenectomy (bilateral inguinal lymphadenectomy, pelvic lymphadenectomy) in the United Kingdom and the United States [10–12]. Radical vulvectomy and the continuing series of skin incisions leading to bilateral inguinal lymphadenectomy (en bloc type) have been vital in enhancing the understanding of dissection and other skills necessary for operating on vulvar cancer and are basic procedures in the vicissitudes of subsequent procedures (CQO2). In recent years, as increased focus has been placed on postoperative QOL, efforts have been made to improve QOL through the individualization and reduction of the two components of radical vulvectomy, namely surgery on the vulvar disease and surgery on the lymphatic nodes [13, 14] (CQ03, CQ05). The results of detailed histopathological examinations of metastasis to the inguinal lymph nodes and risk factors for prognosis have provided the theoretical basis for the reduction and individualization of surgical therapies [15–18]. Moreover, the appropriateness of reduction has been confirmed in meticulous reviews of surgical therapy in early stage vulvar cancer [19]). In inguinal lymphadenectomy, there have been moves toward reduction to superficial inguinal lymphadenectomy alone, but deep inguinal lymphadenectomy is still thought to be necessary based on the results of a prospective clinical trial and recent research on sentinel lymph nodes (SLNs) [19] (CQ05).

In the field of gynecology, the first disease adopting the SLN biopsy procedure was vulvar cancer [20] (CQ06). There have been several reports of prospective clinical trials and reviews [21–24]. From these results, it has been pointed out that omitting the complete removal of the lymph nodes when no metastasis is observed in the sentinel nodes clearly reduces postoperative complications and prognostic symptoms, thereby contributing to improved postoperative QOL. The problem is that safety must be secured by minimizing failure to detect metastasis. Fortunately, it has been confirmed that the relapse rate is low when the target of the surgery is restricted to tumors that are clinically metastasis-negative and localized to the vulva. In addition, it is emphasized that this procedure must be performed by highly trained surgeons and teams [22–24].

The usefulness of extensive surgery resecting surrounding affected organs has long been recognized in cases of advanced vulvar cancer [25]. In advanced cases of vulvar cancer in which the lesion extends from the vagina to the urinary bladder or to the anus and/or rectum, anterior, posterior or total pelvic exenteration is selected. Because of the wide range of defects, various forms of reconstructive surgery must accompany this procedure. Reduced QOL and frequent complications are problems with this procedure, and in recent years reports and reviews have cited the use of preceding radiation therapy and chemotherapy as a means of avoiding extensive surgery, and these have been reported as useful [26] (CQ04).

When large parts of the vulva and surrounding area are lost as a result of vulvar surgery, it is useful to accompany with reconstructive surgery. By making proactive use of cosmetic reconstructive surgery, it has become possible to reduce postoperative complications, improve QOL and limit impairment of the patient’s appearance. A wide range of methods of reconstructive surgery are available, and reconstruction can be tailored to each excision wound, so careful examination is in order before proceeding with surgery [27].

Most patients are elderly and medical complications are frequent. Surgeons preparing to proceed with vulvar surgery must become experts in postoperative management and management of the possible complications, particularly excision infection and dehiscence of operative wounds.

(3) Radiation therapy

The principal role of radiation therapy is as adjuvant therapy to surgery (CQ07). In GOG37, postoperative irradiation of the groin and pelvis is reported to be effective if lymph node metastasis is clinically recognized, if metastasis to the inguinal lymph nodes is combined with fixation or ulceration, or if two or more lymph node metastases are recognized histopathologically [28]. Retrospective research also reports that postoperative irradiation is effective against vulvar primary lesions in cases with positive or close margins [29].

Because patients with vulvar cancer are typically elderly, surgery is not applied in some cases because of medical complications. Definitive radiation therapy is applied in these cases, as well as in inoperable cases with locally advanced cancer (CQ07) [30–32].

Clinical trials have been conducted using preoperative irradiation on locally advanced vulvar cancer (CQ07). In four phase II trials examining the efficacy of preoperative irradiation in cases of stage II−IVa disease and in cases of local recurrence, clinical complete remission was observed in 27–64% of cases, while histopathological complete remission was obtained in 31–70% of cases [30, 33–35]. While these results indicate the effectiveness of multidisciplinary therapy aiming to preserve the function of adjacent organs, problems of consistency still remain. The treatment method is not uniform and criteria for evaluating operability have not been demonstrated. At present, clear evidence is still lacking to support the application of preoperative irradiation to locally advanced vulvar cancer as a standard method.

Three-dimensional conformal radiation therapy (3D-CRT) is a standard method of the external beam radiation therapy. However, intensity-modulated radiotherapy (IMRT) is a more useful treatment that can deliver a conformal radiation dose to a target extending from the vulva to the inguinal region and pelvic lymph nodes.

(4) Chemotherapy

Reports of chemotherapeutic treatment of vulvar cancer have increased in recent years. However, all the studies were at the phase II trial level, and no standard treatment has been established (CQ08, CQ10). Chemotherapy is selected in the following situations including preoperative chemotherapy, CCRT, postoperative adjuvant chemotherapy, and chemotherapy against progression and relapse.

### Clinical questions and recommendations

#### Surgical treatment

CQ 01: What treatments are recommended for VIN?

Objective

The objective is to determine a treatment method for VIN.

Recommendations

(1) Periodic follow-up is recommended for LSIL (Grade A).

(2) Wide local excision, simple vulvectomy, or laser vaporization may be considered, depending on the case, or laser vaporization may be combined with either of the first two for HSIL or dVIN (Grade C1).

Comments

Many reports about VIN have been published in accordance with the stage categories VIN 1 to VIN 3, advocated in the WHO classification 2003 (3rd edition). However, the latest WHO classification identifies three new categories as mentioned in general consideration. LSIL and HSIL occur in relatively young patients, have been increasing in recent years, and represent the majority of VIN cases. Although most cases of LSIL regress spontaneously, 6% of HSIL cases have been shown to progress to squamous carcinoma [36, 37]. BP, which most commonly occurs in patients in their late teens and 20s, presents histopathologically as HSIL, but frequently regresses spontaneously without any treatment [38–42]. On the other hand, dVIN, which is related to lichen sclerosis and lichen planus, corresponds to simplex VIN in the old WHO classification. Simplex VIN often occurs in elderly patients and clinically presents as leukoplakia vulvae. It has been pointed out that simplex VIN progresses to squamous carcinoma in 33% of cases, and is more malignant than LSIL or HSIL [37, 43]. Because LSIL, HSIL and dVIN differ in pathogenesis and malignancy, it is preferable that each be managed differently.

Most cases in the old VIN 1 category are LSIL, and doubt exists as to their significance as neoplastic lesions. However, the old histopathological definition of VIN 1 includes dVIN which, unlike LSIL, is a neoplastic lesion and must be separated from VIN 1 even though the frequency of dVIN occurrence is very low [44]. On that understanding, it is preferable to avoid invasive treatment for LSIL and to follow-up periodically. On the other hand, HSIL and dVIN are neoplastic lesions which require treatment. In a systematic review of the literature, it was found that 9% of untreated cases of VIN 3 progressed to invasive carcinomas, and 3% of cases that are surgically excised had occult invasive carcinoma [45], indicating that biopsy under colposcopy is important to exclude invasion [46]. Clinically the evidence for a clear distinction between VIN 2 and VIN 3 has not been demonstrated, and HSIL, which includes both VIN 2 and VIN 3, must be managed with the same due consideration [7, 36]. Because HSIL is caused by HPV infection, multiple foci of disease can appear in a wide-ranging area of the vulva and can also appear, simultaneously or allochronically, in and around the uterine cervix, vagina and anus. Careful examination of all of these areas is required.

When part of a VIN lesion is suspicious for invasion on the appearance of ulceration or irregular surface, it is necessary to move proactively to carry out a wide local excision or simple vulvectomy and conduct a histopathological examination on excised specimens, even if biopsy results do not identify the invasion. In particular, dVIN is often complicated with, or progresses to, invasive carcinoma, and surgical excision is the first choice of treatment that should be considered. In surgical procedures, physicians should consider the patient’s QOL and carry out shallow excision, avoiding deep excision of the vulva, and take care in preserving the clitoris [47, 48]. If the range of excision is very wide, cosmetic surgery of the vulva using skin grafts may be added [7].

If a case of HSIL or dVIN has been comprehensively confirmed not to be associated with invasion, it is possible to choose laser vaporization instead of surgical excision [49]. If the focus of disease is wide, or if there are multiple foci, it is possible to treat the condition on a case-by-case basis with a combination of vaporization and surgical excision. However, lesions clinically judged to be BP have been reported to regress spontaneously over a period of 3–30 months (median period 9.5 months) [39]. If the focus of disease is not seen to regress under strict periodic follow-up, treatment should be administered.

Recently imiquimod ointment has been used to treat LSIL and HSIL to stimulate local immune response [40, 50–54]. Effectiveness for this approach has been indicated by prospective controlled trials and meta-analyses. However, no randomized trial has compared imiquimod treatment to standard treatment, and the evidence remains insufficient due to the small number of cases. Imiquimod treatment is not a standard treatment at present and is not listed as an insured treatment in Japan.

Currently, a preventive vaccine for HPV is expected to show effectiveness against HPV-related lesions, and a decline in cases of LSIL and HSIL is anticipated as adoption of the vaccine spreads [55].

Because of the high frequency of recurrence and the possibility of transition into vulvar cancer, periodic follow-up is an important component of management of intraepithelial lesions [56].

CQ 02: How should radical vulvectomy be applied? What techniques should be used?

Objective

The objective is to examine how radical vulvectomy should be applied as a curative excision and the techniques that should be used.

Recommendations

(1) Radical vulvectomy is recommended in cases where the focus of disease is localized to the vulva or perineum when the diameter of the tumor is >2 cm and the stromal invasion is >1 mm deep (Grade B).

(2) Resection of vulvar tumors and inguinal lymph nodes through separate incisions is recommended (Grade B).

Comments

Until the first half of the 20th century, surgical methods for vulvar cancer (squamous carcinoma) were limited to vulvectomy in advanced cases. In those days, the 5-year survival rate was 20–25%. Later, the survival rate improved to >60% with the introduction of en bloc incision, in which excision was applied to a continuous mass incorporating the skin and subdermal tissue of the vulva and the adipose tissue of the groin, including the inguinal lymphatic nodes, and combined sometimes with removal of the pelvic lymphatic nodes [10, 11]. This combination of radical vulvectomy plus inguinal and pelvic lymphadenectomy became the standard surgical procedure. However, many cases were accompanied by special site characteristics, very elderly patients, or various medical complications. As a result, the surgery was associated with significant morbidity, including wound dehiscence and infection [57]. In the 1980s, it was reported that postoperative complications could be reduced through conservative surgery, and the focus began to shift toward individualized treatment. However, because of the many sites of occurrence and foci of diseases in individuals of vulvar cancer and the low frequency of occurrence, many reports gathered cases over a period of >20 years. There were no randomized controlled trials of incision methods, and clear evidence of the effect of conservative surgery on vulvar cancer was limited. Complicating matters further was the lack of reports based on the new FIGO classifications; the existing literature was based entirely on the 1988 FIGO classifications. Therefore, radical vulvectomy should be applied in cases where application of conservative surgery was not clearly indicated, i.e., the tumor is on the median, on the side of the pubic bone, the tumor is on both the left and right sides, and multiple foci of disease are present.

As an improvement to radical vulvectomy, separate incision (or triple incision) was reported in 1962. In this approach, the excision of vulvar neoplasia is separated from inguinal lymphadenectomy [58]. Later, in the 1980s, a method was developed to leave the suprapubic skin intact as a skin bridge. The prognosis for this procedure was found to be commensurate with historical data in stages I–IV in the old (1988) FIGO classifications, and the frequency of wound complications decreased dramatically [59–62]. In particular, a comparative matched study was conducted on 32 patients per group with squamous cell carcinoma localized to the vulva and perineum, where the tumor was ≤2 cm in diameter and with lesions >2 cm,in which either en bloc incision or separate incision was performed. While overall survival and disease-free survival rates were similar for the two groups, the dehiscence rate for vulvar and inguinal wounds decreased significantly in the case of separate incision [63]. Separate incision was shown to be clearly less surgically invasive than en bloc incision. Although skin bridge recurrence between the vulva and groin was higher for separate incision than for en bloc incision, the survival prognosis after re-excision was good [64, 65]. Furthermore, cases of skin bridge recurrence between the vulva and groin are <1% where no gross lymph node metastasis is present. Although recurrence in lymph nodes is lower for en bloc incision, no difference in survival prognosis is found. Currently, even when the frequency of recurrence in lymph nodes and skin bridge recurrence between the vulva and groin is considered, separate incision is recommended, due to the lesser degree of impairment caused by the treatment [66].

When vulvar disease invades the urethral opening or lower urethra, it is possible to excise the urethra in a way that preserves the urethral sphincter and does not cause incontinence [67]. Invasion to the vagina as far as the lower third of the vaginal wall can also be excised at the same time as vulvectomy.

The term ‘radical vulvectomy’ also includes the modified radical vulvectomy which preserves part of the healthy vulva.

CQ 03: How should conservative surgery be applied for invasive vulvar cancer?

Objective

The objective of this section is to consider methods of conservative surgery planned for early stage vulvar cancer and how to apply them.

Recommendations

(1) Wide local excision is recommended in cases where the diameter of the tumor is ≤2 cm and the stromal invasion is ≤1 mm deep (Grade B).

(2) Radical local excision can be considered with an excision margin of 2 cm in cases where the diameter of the tumor is ≤2 cm but the stromal invasion is >1 mm deep; or the diameter of the tumor is >2 cm but the lesion is localized laterally to the vulva or to the perineum (Grade C1).

Comments

In early stage vulvar cancer, the size of the tumor and depth of invasion are related to metastasis to the inguinal lymph nodes. If the diameter of the tumor is ≤2 cm and the stromal invasion is ≤1 mm deep, wide local excision is an appropriate surgical method that offers low surgical invasiveness, and inguinal lymphadenectomy can be omitted [68–71].

Radical local excision may be considered in cases where the diameter of the tumor is ≤2 cm but the stromal invasion is >1 mm deep; or the diameter of the tumor is >2 cm but the lesion is secluded (localized laterally to the vulva or to the perineum), with surrounding skin tissue normal. This surgical technique presents a considerably lower frequency of postoperative complications than radical vulvectomy, and while the local recurrence rate is reported to be somewhat elevated, no difference is observed in the survival period [72–75]. The procedure should be limited to tumors which occur singly and are lateral (defined as ≥1 cm distant from the median line of the lesion).

In this procedure the depth of the excision is similar to that of a radical vulvectomy. The excision margin is closely related to the probability of local recurrence; if the margin is ≤8 mm, the probability of local recurrence is 50%. The local control rate is high when the margin is secured for 8 mm to 1 cm histopathologically [76, 77]. However, even if the excision margin is a gross 1 cm, histopathologically the excision margin is ≤8 mm after fixation in 50% of cases. To ensure sufficient excision margin, a gross distance of 2 cm is required [64].

CQ 04: What surgical therapies are recommended for locally advanced cases with adjacent organs infiltrated?

Objective

The objective of this section is to examine the usefulness of pelvic exenteration in locally advanced cases with deep adjacent organ invasion.

Recommendations

(1) Pelvic exenteration is considered if there is no apparent lymph node metastasis and complete excision is anticipated (Grade C1).

(2) Preoperative CCRT or chemotherapy is also considered in order to avoid QOL decline associated with pelvic exenteration (Grade C1).

Comments

In the past, pelvic exenteration was considered as an option for locally advanced vulvar cancers deeply infiltrating adjacent organs, such as the urethra, urinary bladder, anus and rectum. According to a retrospective study on 19 cases of vulvar squamous carcinoma receiving pelvic exenteration, including 11 cases of primary treatment, the 5-year survival rate was 60%. Although no difference was found in overall survival between primary treatment and recurrent cases, lymph node metastasis was significantly associated with overall survival [78]. In a retrospective study of 27 cases of stage III−IV vulvar cancer (FIGO 1994) receiving pelvic exenteration, including 9 cases of primary treatment, the 5-year survival rate was 62% [25]. Although no significant difference in prognosis was found between primary treatment and recurrent cases, cases with negative lymph node metastasis had significantly better prognoses than positive cases. Furthermore, cases of pathologically confirmed complete excision had significantly better prognosis than cases of pathological incomplete excision. These results indicate that the presence/absence of lymph node metastasis and complete excision are the most important prognostic factors [25].

In recent years, conservative surgery preceded by chemotherapy or CCRT has been tried as an alternative to pelvic exenteration, in order to avoid compromising QOL. According to a retrospective study and Gynecologic Oncology Group (GOG) phase II trials, preoperative CCRT can enable cases that are unresectable or requiring pelvic exenteration to be resectable or to circumvent pelvic exenteration [30, 34, 79]. However, since no phase III trials exist to date, the prognostic impact remains unclear.

**Fig. 2:**
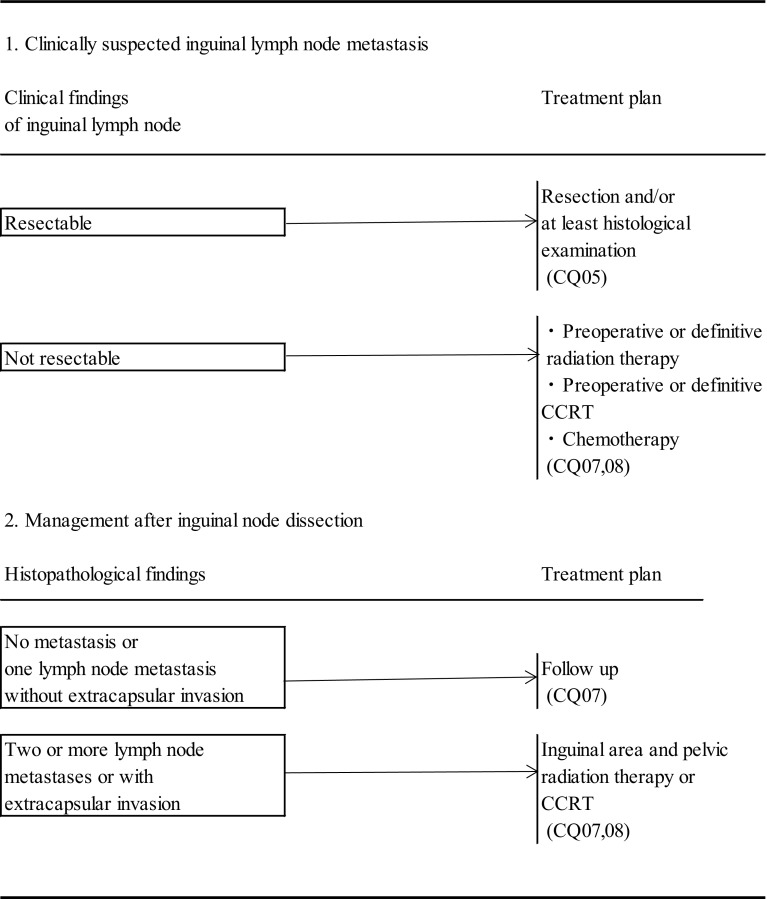
Management of inguinal lymph nodes of vulvar cancer

CQ 05: How and in what range should lymphadenectomy be applied? (Fig. 2)

Objective

Although the superficial inguinal and deep inguinal lymph nodes are the regional lymph nodes affected by vulvar cancer, efforts have been made to reduce the scope of lymphadenectomy according to the disease state. The objective of this section is to examine the usefulness and appropriate scope of lymphadenectomy.

Recommendations

(1) Inguinal lymphadenectomy can be omitted in cases where the tumor is ≤2 cm in diameter and the depth of the stromal invasion is ≤1 mm (Grade B).

(2) Inguinal lymphadenectomy includes resection of both the superficial and deep inguinal lymph nodes (Grade C1).

(3) Ipsilateral lymphadenectomy alone can be considered in cases of a tumor located laterally in which the tumor is ≤2 cm in diameter (Grade C1).

(4) Postoperative radiation therapy for the groin and pelvis is recommended in cases that are positive for metastasis in the inguinal lymph nodes, where radical vulvectomy and inguinal lymphadenectomy have been conducted (Grade B).

(5) It is recommended at a minimum that the enlarged lymph nodes be resected if possible, to conduct a histological examination for possible metastasis (Grade B).

Comments

Prognosis is extremely poor with recurrence in the inguinal lymph nodes in cases of vulvar cancer in which inguinal lymphadenectomy was not performed. To ensure elimination of lymph node metastasis (the most important factor in prognosis), it is considered essential to conduct inguinofemoral lymphadenectomy on at least one side during primary radical surgery [80, 81]. In randomized controlled trials (GOG88) of radiation therapy to the groin versus lymphadenectomy (postoperative irradiation of the diseased-side groin and pelvis in cases positive for lymph node metastasis) in cases in which metastasis to the inguinal lymph nodes is not suspected, recurrence was higher in the radiation therapy group than in the lymphadenectomy group. Both progression-free survival time and overall survival time were significantly better for the inguinal lymphadenectomy group, prompting the researchers to discontinue the trials. These trials suggest that lymphadenectomy is more useful than radiation therapy to the groin in the treatment of vulvar cancer with no suspicion of inguinal lymph node metastasis [82].

However, in cases where the tumor is ≤2 cm in diameter and invasion is ≤1 mm deep (corresponding to clinically stage IA), lymph node metastasis occurs in <1% of cases. Lymphadenectomy is not recommended in these cases, as this treatment is ineffective and in fact harmful [83]. Even in cases where invasion is >1 mm, omission of lymphadenectomy is reported based on considerations, for example, on the presence/absence of lymphovascular space invasion, lesion sites and the degree of histological differentiation [84]. Further evaluation of criteria for omission of lymphadenectomy in these cases is warranted in the future.

Cases have been reported in which no recurrence is confirmed when treatment consists of removal of the superficial inguinal lymph nodes alone, if the tumor is ≤1 cm in diameter and invasion is ≤5 mm deep [68]. Later, retrospective studies were conducted for cases with early stages that were treated with removal of the superficial inguinal lymph nodes alone. Recurrence was confirmed on the same side in only 4% of cases. This result indicates that treatment consisting solely of superficial inguinal lymphadenectomy is effective [74]. However, in a prospective one-arm trial of GOG74, superficial inguinal lymphadenectomy alone was conducted in cases in which the tumor was ≤2 cm in diameter, no vascular space invasion was present, lymph nodes were not swollen and invasion was ≤5 mm deep. In comparison with past research results [81, 84], no difference in survival rate was found, but the recurrence rate was high (16%) and recurrence in the treated lymph nodes side was confirmed in six of 121 cases [85]. According to later, retrospective studies, the recurrence rate was also higher for superficial inguinal lymphadenectomy alone compared with lymphadenectomy as far as the deep inguinal lymph nodes [86, 87]. Another study points out that the SLNs are superficial inguinal lymph nodes in 84% of cases and deep inguinal lymph nodes in 16% of cases, indicating that in some cases the deep inguinal lymph nodes are the SLNs [88]. These findings suggest that lymphadenectomy should be extended as far as the deep inguinal lymph nodes.

On the question of whether bilateral inguinal lymphadenectomy is necessary, the organs in which bilateral lymph flow is observed anatomically consist of the perineum, clitoris, and labia minora near the pubis [89]. Retrospective studies indicate that when the focus of disease is ≤2 cm in diameter and is located on one lateral side, metastasis to contralateral lymph nodes occurs in <0.5% of cases [83, 90]. These findings suggest that lymphadenectomy can be limited to the diseased side in cases where the tumor is ≤2 cm in diameter, has not invaded to the midline structures such as the clitoris, urethra, vagina, perineal body or anus, is 1–2 cm lateral from the median, and no lymph node metastasis is suspected. However, some reports indicate that bilateral lymphadenectomy is necessary if lymph node metastasis has occurred on the diseased side [85]. Another study reports that metastasis to the non-diseased side is possible only in cases where the tumor is ≥2 cm in diameter, invasion is ≥5 mm deep, and ipsilateral lymph node metastasis is confirmed. In these cases, bilateral lymphadenectomy is recommended [91]. On the question of whether bilateral inguinal lymphadenectomy is necessary for all medial lesions, some reports indicate that metastasis to the opposite side is not confirmed in cases where the SLNs are identified on only one side [92]. As with the problems of superficial and deep inguinal lymphadenectomy, the answer would be changed in the future according to the generalization of SLN biopsy.

To examine the question of additional treatment for positive cases of metastasis to inguinofemoral lymph nodes, randomized controlled trials (GOG37) were conducted comparing pelvic lymphadenectomy with radiation therapy to the pelvis and groin after radical vulvectomy and full inguinal lymphadenectomy. It was reported that radiation therapy was superior in terms of 2-year overall survival rate. The addition of radiation therapy significantly reduced local recurrence and cancer-related death, according to follow-up data over a median period of 74 months. As a result, treatment with pelvic lymphadenectomy with no radiation therapy was not recommended [93]. However, some guidelines indicate that all of the swollen inguinal and pelvic lymph nodes may be removed and followed by radiation therapy to the groin and pelvis in cases where imaging revealed swelling of the pelvic lymph nodes. As such, surgery to remove the pelvic lymph nodes is by no means repudiated [94].

One retrospective comparative study examined how to handle swollen inguinal lymph nodes with suspicion of metastasis, comparing removal of the swollen lymph nodes alone with systematic lymphadenectomy. It was found that the selection of limited surgery had no harmful effect on prognosis, provided it was coupled with appropriate postoperative irradiation [95]. Although the study did not draw any conclusions regarding the necessity of systematic lymphadenectomy, it seems likely that at least the excision of swollen lymph nodes suspected of metastasis should be considered, and the presence/absence of metastasis examined histologically, before considering radiation therapy.

CQ 06: Can SLN biopsy be used to avoid performing a lymphadenectomy?

Objective

The objective of this section is to examine whether systemic inguinal lymphadenectomy can be avoided by using SLN biopsy.

Recommendations

SLN biopsy may be performed as a means to avoid an inguinal lymphadenectomy in cases in which metastasis to the inguinal lymph nodes is not suspected clinically. In view of the current status of experience in the procedure in Japan, this action should be considered as an experimental approach (Grade C1).

Comments

The identification of SLNs for vulvar cancer is one of the most progressed areas of inquiry in gynecology [96, 97]. In principle, SLN biopsies are used in cases with vulvar cancer localized to the vulva and perineum with no suspicion of lymph node metastasis.

According to numerous reports from individual institutions, there are three methods of identifying the SLNs—the dye method alone, the radioisotope (RI) method alone, and the dye/RI combined method [98, 99]. Isosulfan blue and patent blue are used in the dye method, and technetium-99 m is used in the RI method. Recently, a feasibility study has been reported of a fluorescence method using indocyanin green dye in cases of vulvar cancer [100]. An analysis of the combined method based on 26 journal articles and 1271 cases indicates that this method provides an 86% identification rate and a false negative rate of 5.8%. For the RI method, analysis of seven articles and 116 cases yields an identification rate of 83% and a false-negative rate of 8.8%. For the dye method alone, analysis of three articles and 111 cases provides a low identification rate of 64% and a false-negative rate of 8.7%. Most reports recommend use of the combined method [98].

According to a meta-analysis, the following factors affect the identification rate for SLNs and the sensitivity for metastasis—method of identification of SLNs, and localization of the focus of disease in the vulva, i.e., whether the focus of disease is located medially or laterally. An increase in false negatives for medial lesions has been reported [99]. A multi-institutional validation study similarly report false negatives for medial lesions [21]. Similarly, one meta-analysis indicated a decrease in identification rate for SLNs in cases with palpable swollen lymph nodes or with large tumors [99].

In a multi-institutional prospective study, SLN biopsies were used to avoid systemic lymphadenectomy when the SLNs were negative for metastasis. In the trial, 403 cases of tumors <4 cm in diameter and localized in the vulva were examined. The recurrence rate in the inguinal lymph nodes was 3%, indicating the effectiveness of the SLN biopsy. In particular, the recurrence rate was 2.3% in isolated vulvar disease [22]. Complications of SLN biopsies such as wound trouble, cellulitis and lymphedema were rare in the study [101].

In a cost-effectiveness analysis, omission of lymphadenectomy based on SLN biopsy was compared with systemic inguinal lymphadenectomy. This report found that the most cost-effective approach was to identify SLNs using the combined method (dye plus RI) and perform ultrastaging using immunostaining to search for metastasis [102]. In the multi-institutional trials cited above, the metastasis detection rate was found to increase gradually when immunostaining was combined with multi-section searches (ultrastaging) using ordinary hemotoxylin-eosin (H&E) dye [22]. However, the intervals in which sections were prepared and the number of sections searched were not consistent in the reports.

Frozen section diagnosis during surgery is reported as useful, with a sensitivity of 89% and a negative predictive value of 93% [103]. However, the sensitivity and negative predictive value were both low at 48 and 78%, respectively, in a multi-institutional trial [22]. Opinion differs as to whether this practice should be combined with postoperative ultrastaging or omitted when searching for metastasis in SLNs.

Even though evidence was accumulated for the usefulness of SLN biopsy in vulvar cancer, no results of randomized controlled trials currently exist comparing this approach with standard treatments. In Japan, almost no results are available on vulvar cancer, as health insurance covers SLN biopsies only for breast cancer and malignant melanomas. Under these conditions, the first step should be to conduct clinical trials on the omission of lymphadenectomy on the basis of SLN biopsies. It is also preferable that the omission of systematic lymphadenectomy be carried out not by gynecologists alone but with the cooperation of physicians in other medical fields who are thoroughly trained in SLN biopsies, as well as radiologists, pathologists and others.

#### Radiation therapy for vulvar cancer

CQ 07: How should radiation therapy be applied and what methods should be used?

Objective

Radiation therapy is conducted as an adjuvant treatment to surgery and as a definitive treatment. The objective is to examine how radiation therapy should be applied and the methods that should be used.

Recommendations

(1) Postoperative irradiation of the primary site may be considered if the excision margin is <8 mm, or advanced vascular space invasion is confirmed (Grade C1).

(2) Postoperative irradiation of the groin and pelvis is recommended, if two or more metastases or extracapsular invasion of lymph node metastasis is confirmed in the inguinal lymph nodes (Grade B).

(3) Omission of postoperative irradiation may be considered, when one metastasis alone of the inguinal lymph nodes has occurred without extracapsular invasion (Grade C1).

(4) Definitive radiation therapy may be considered in inoperable cases (Grade C1).

(5) Preoperative irradiation may be considered as a means of preserving the function of adjacent organs in locally advanced cases (Grade C1).

(6) Concurrent chemotherapy with a single platinum-based drug or combination of this drug may be considered (Grade C1).

Comments

Postoperative recurrence of vulvar cancer is associated with the degree of tumor invasion and lymph node metastasis [76, 81, 104]. Because the risk of local recurrence appears if excision margins are <8 mm [76], postoperative adjuvant treatment for the primary site is required. In a retrospective study, postoperative irradiation of the primary site is reported to be effective in cases with close margins or where advanced vascular invasion is recognized [29]. As mentioned in CQ05, randomized controlled trials (GOG37) have demonstrated the superiority of postoperative irradiation to pelvic lymph node dissection when inguinal lymph node metastasis is recognized, when lymph node metastasis has occurred in combination with fixation or ulceration, or when two or more lymph node metastases are confirmed histopathologically [28, 93]. One retrospective study of 208 cases of single lymph node metastasis found that postoperative irradiation was effective in increasing disease-specific survival rate. Unfortunately, this study is problematic as it fails to consider extracapsular invasion in lymph node metastasis, which is a major risk factor for recurrence [105]. Later, a multi-institutional retrospective study was conducted on 75 cases of single lymph node metastasis without extracapsular invasion. This study did not confirm that postoperative irradiation was beneficial [106]. Based on these findings, inguinal and pelvic postoperative irradiation is recommended when two or more metastases are confirmed in the inguinal lymph nodes, or extracapsular invasion is confirmed. In practice, the field of pelvic lymph nodes covers the internal and external iliac lymph nodes and the obturator lymph nodes, and the radiation dose is 45–50.4 Gy in 25–28 fractions.

Definitive radiation therapy is considered in cases where surgery is not applicable, such as in elderly patients or in the case of medical complications, and in cases that are judged inoperable, such as those with advanced local progression or metastasis in inguinal lymph nodes that cannot be removed. When planning radiation therapy, the groin is included in the clinical target volume (CTV) even if no metastasis is clinically confirmed, and the CTV is extended to the pelvic lymph nodes if metastasis is clinically confirmed in the inguinal lymph nodes. After external beam irradiation of 45–50 Gy of a CTV encompassing the lymph node area, the irradiation field is shrunk to the focus of disease and the dose is increased to 60–70 Gy. Because it is necessary to apply a uniform dose to a complex CTV that includes the vulva, groin and pelvic lymph nodes, 3D-CRT combining X-ray and electron beam irradiation is applied. Compared with conventional 3D-CRT, IMRT enables more conformal dose distribution to the target and reduced dose on surrounding normal organs [32].

Some reports have examined the usefulness of multidisciplinary therapy using preoperative irradiation on tumors that are inoperable due to invasion of adjacent organs, or in cases of locally advanced vulvar cancer requiring exenterative surgery. In the results of four phase II trials targeting stages II, III, IVA and cases in local recurrence, clinical complete remission was confirmed in 27–64% of cases, 72–97% of cases became operable, and histopathological complete remission was obtained in 31–70% of surgical cases [30, 33–35]. Although the scale of the trials was small (41–71 cases), the results of each trial indicated the effectiveness of multidisciplinary therapy aimed at preserving the function of adjacent organs. However, the criteria for application of surgery remain unclear; QOL evaluation is not always performed, and the degree to which histopathological complete remission improves prognosis is not clearly understood [107]. Furthermore, no phase III trials have been conducted indicating the effectiveness of preoperative irradiation on advanced local vulvar cancer [26]. Accordingly, physicians must exercise careful judgment before applying preoperative irradiation, even with the aim of preserving organ function.

Many recent reports indicate the concurrent use of cisplatin (40 mg/m^2^/week), and adverse events were found to be within the acceptable range [30–32]. If the patient’s general condition and organ function meet certain criteria, the use of concurrent chemotherapy based on platinum-based drugs may be considered.

The most common acute radiation morbidity is radiation dermatitis, which occurs in virtually all cases. In some cases this condition becomes serious or is combined with infection, requiring the suspension or termination of treatment [108]. Careful management is required, giving due consideration to protection of the skin and prevention of infection during radiation therapy.

#### Chemotherapy for the vulvar cancer

CQ 08: How should chemotherapy be applied?

Objective

The purpose of this section is to examine the usefulness of chemotherapy as a treatment for vulvar cancer.

Recommendations

(1) Preoperative chemotherapy may be considered for locally advanced cases (Grade C1).

(2) Systemic chemotherapy may be considered for cases of progression or recurrence of distant metastasis (Grade C1).

Comments

In contrast to the effectiveness of radiation therapy in the treatment of advanced vulvar cancer, there are few reports for chemotherapy which has been regarded as ineffective. Even so, attempts have been made to use chemotherapy as a preoperative adjuvant treatment for advanced cases to make them operable. Chemotherapy is also expected to be beneficial in advanced cases with distant metastasis and in recurrent cases.

Primary surgery of locally advanced vulvar cancer requires pelvic exenteration or other extended surgical methods. These surgeries result in impaired QOL. To address this problem, efforts have been made to use preoperative chemotherapy in advanced cases of vulvar cancer to avoid extended surgery. Many reports on these attempts have been generated from European organizations such as the European Organization for Research and Treatment of Cancer (EORTC). In these trials, preoperative chemotherapy was tested on locally advanced vulvar cancer using drugs such as bleomycin, methotrexate, lomustine, cisplatin, 5-fluorouracil, paclitaxel and vincristine. The reported response rate and surgical completion rate after chemotherapy were both favorable at approximately 60% and 57–90%, respectively [109–113]. In each report, however, the number of cases examined was small, and no standard treatment regimen was established. Problems remain with respect to the selection of drugs.

In phase II trials, the response rate of preoperative chemotherapy was higher than that of chemotherapy usually applied to advanced and recurrent cases. In most cases of recurrence, the patient has undergone radiation therapy in the past, and the low response rate may be found in advanced and recurrent cases for chemotherapy. The reported response rate for treatment with cisplatin alone [114], mitoxantrone [115] and a weekly dose of paclitaxel plus carboplatin [116] is 0% in each case, while for paclitaxel alone the response rate is 14% [117]. In more encouraging treatment results, the response rate for bleomycin alone or concurrent with mitomycin, reported in 1980, was 50% [118], while a response rate of 40% is reported for concurrent treatment with cisplatin and vinorelbine [119]. Because no studies have found reproducibly high response rates of chemotherapy, no treatments could be regarded as the standard treatment. However, in advanced cases with distant metastasis and in recurrent cases after radiation therapy, chemotherapy may be the only treatment option. Trials are currently under way using a wide variety of drugs [120].

Although the most widely used postoperative adjuvant treatment for vulvar cancer is radiation therapy, the use of chemotherapy as a postoperative adjuvant treatment is also reported [121]. In this report, cisplatin was used postoperatively as adjuvant treatment in 14 cases of vulvar cancer with lymph node metastasis, and the recurrence rate was low. However, no other reports of similar results exist, and postoperative adjuvant chemotherapy has not become a standard treatment for vulvar cancer.

#### Follow-up and treatment for recurrent diseases

CQ 09: How should periodic follow-up be conducted after treatment?

Objective

The purpose is to examine the intervals and methods by which appropriate periodic follow-up should be conducted regarding recurrence and complications associated with treatment.

Recommendations

(1) A rough guide for the intervals in periodic follow-up after treatment is as follows (Grade C1):

First and second years: every one to three months

Third to fifth years: every six months

Sixth and subsequent years: once a year

(2) Conduct medical interviews, inspection, palpation, cytology, biopsy, chest X-ray, tumor markers and computed tomography (CT). Monitor not only for recurrence but also for complications (Grade C1).

Comments

The purpose of periodic follow-up is to detect recurrence as soon as possible, improve the prognosis, and to mitigate and reduce the loss of QOL caused by treatment. However, no evidence exists that diagnosis of recurrence through periodic follow-up after treatment leads to improved prognosis in vulvar cancer. Moreover, no reliable research has been performed regarding the interval of periodic follow-up and items examined during periodic follow-up after treatment of vulvar cancer, and no consensus can be obtained.

Guidelines in countries other than Japan were reviewed. In the United Kingdom, the Royal College of Obstetricians and Gynecologists recommends observation every 3 months during the first year after treatment, every 6 months in the second and third years, and once every 12 months in the fourth and subsequent years [122]. In contrast, the National Cancer Institute (NCI) of the United States has no recommendations regarding periodic follow-up [123]. The recommendations are based on the results of retrospective studies indicating that local recurrences diagnosed at a routinely scheduled follow-up were found to have a smaller greater dimension compared with recurrences detected by chance [124]. However, no significant effect on survival rate was confirmed. In retrospective studies examining the timing, site of recurrence and the prognosis, 55–67% of recurrences occurred within 2 years after the first surgery [125, 126]. The most important predictive factors for recurrence are stage and metastasis to the inguinal nodes [125–127]. In cases that are positive for metastasis to the inguinal nodes at the time of initial treatment, recurrence within the first 2 years is common. On the other hand, in cases that are negative for metastasis to the inguinal nodes at the time of initial treatment, the recurrence rate is low, and recurrence sites are mainly local [125].

Because the common sites of recurrence are the local site and the inguinal nodes, inspection and palpation of these sites are most important. If recurrence is suspected, cytology and biopsy should be conducted. Other useful examination items are chest X-ray, tumor markers, CT, magnetic resonance imaging (MRI) and 2-deoxy-2-[^18^F]fluoro-d-deoxy glucose-positron emission tomography (FDG-PET). No set criteria are established regarding timing or other aspects of these tests. Detailed medical interviews of the patient’s condition enable the physician to make decisions after considering the risk of recurrence in each case. Although recurrence within the first 2 years is common, recurrence also occurs ≥5 years after initial treatment, which demonstrates the need for long-term follow-up [127].

The probability of postoperative complications in vulvar cancer is relatively high [128]. The rate of wound complications of the vulva ranges from 9−58%, while frequent urination or urinary incontinence is confirmed in 8–28% of cases. The frequency of inguinal complications following inguinal lymphadenectomy is reported as 21–39% for infection, 17–39% for wound dehiscence, 21–57% for cellulitis, 11–40% for lymphocele and 14–48% for lymphedema. Most cases of lymphedema occur in the legs within the first 12 months after surgery and are chronic. Lymphedema is aggravated by obesity, infection, addition of radiation therapy and deep vein thrombosis after surgery. Furthermore, psychological and social problems frequently occur after surgery for vulvar cancer, including psychological stress, pain during sexual intercourse, decreased libido, frequent urination and incontinence. Because these problems are detrimental to a patient’s QOL, long-term periodic follow-up is necessary.

**Fig. 3:**
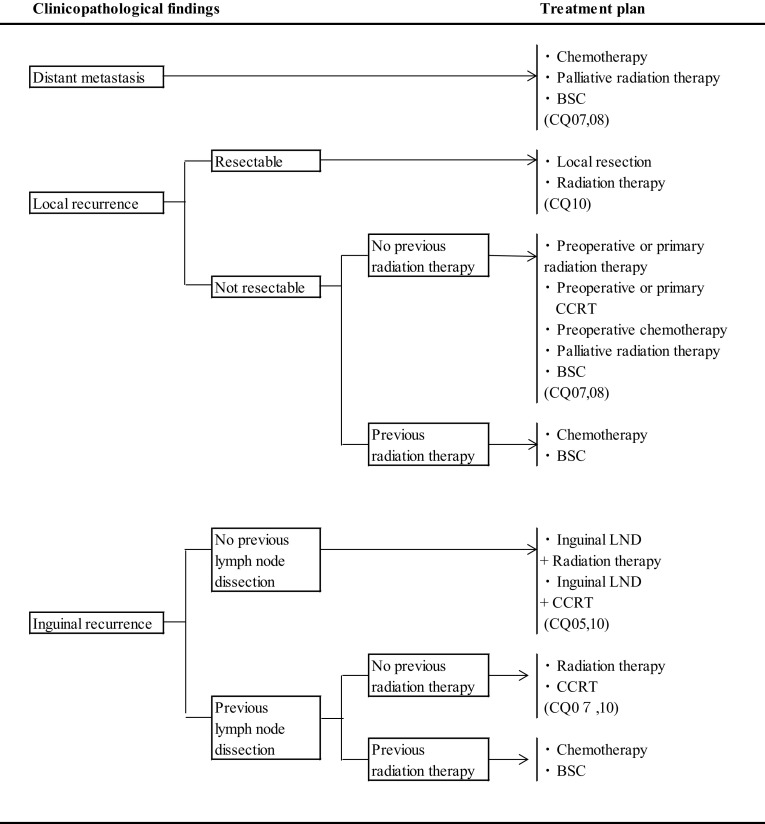
Treatment of vulvar cancer. Treatment of distant metastasis, recurrent tumor

CQ 10: What treatments are recommended for recurrent disease? (Fig. 3)

Objective

The objective of this section is to examine the most useful treatment approaches for recurrence.

Recommendations

(1) Re-excision is considered for postoperative localized recurrence (Grade C1).

(2) CCRT is considered for local recurrence unresectable or infiltrating adjacent organs, if unirradiated (Grade C1).

(3) Systemic chemotherapy is considered for recurrences in the pelvis, with distant metastasis or with multiple lesions (Grade C1).

(4) Best supportive care (BSC) is considered if no other effective treatments are left (Grade C1).

Comments

When deciding treatment for recurrence, the site of recurrence, details of previous treatments and performance status must be taken into account [129]. A retrospective study on 502 cases of vulvar squamous carcinoma in 5 institutions in Italy reported that recurrence occurred in 37% and the 5-year survival rate varied by recurrence site—60% for the vulva, 27% for the groin and pelvis, 15% for distant metastasis, and 14% for multiple lesions [127].

For localized recurrence, re-excision is considered. In a retrospective multi-institutional study of 102 recurrent cases receiving re-excision following radical vulvectomy, the disease-free survival rate was 56% and the 5-year survival rate was 61%. Cases with inguinal lymph node metastasis at recurrence showed significantly poor prognosis [130]. If lymphadenectomy has never been performed, unilateral or bilateral inguinal lymphadenectomy may be performed, depending on the site of recurrence. If the re-excision margin or lymph node metastasis turns out positive, CCRT may be added if radiotherapy has not yet been performed [129].

For local recurrence to unresectable or infiltrating adjacent organs, CCRT is considered if unirradiated. According to a retrospective study where CCRT was performed for 7 unirradiated cases of postoperative local recurrence of vulvar squamous carcinoma which were unresectable or required pelvic exenteration, 4 yielded complete response (CR), 3 yielded partial response (PR), and the 2-year survival rate was 28% [79]. In another study on 15 unirradiated cases of postoperative recurrence which received CCRT, 8 cases yielded CR, and the 2-year survival rate was 46% [131].

Prospective trials also confirm that the prognosis for inguinal recurrence is extremely poor. According to an analysis of two prospective trials (GOG74, GOG88), recurrence occurred in 37 out of 143 cases of initial treatment of vulvar squamous carcinoma. Significantly poor prognosis was demonstrated in 12 cases of inguinal recurrence with a median survival of 9.4 months compared to 20 cases of vulvar recurrence with a median survival of 52.4 months [132]. In cases of inguinal recurrence which have not received lymphadenectomy or radiotherapy, bilateral inguinal lymphadenectomy followed by radiotherapy or CCRT is considered. In cases after external irradiation, interstitial irradiation may be considered, but complications are frequent [129]. Re-excision for inguinal recurrence after irradiation also requires caution because of frequent postoperative complications.

In cases of recurrence in the pelvis, with distant metastasis or with multiple lesions, chemotherapy is considered. Several phase II studies reported on the effects of chemotherapy for recurrent diseases [109, 117, 119]. However, no treatment could be regarded as the standard treatment as previously mentioned in CQ08. Chemotherapy is considered only when no other effective treatments are available.

In recurrent vulvar cancer, most patients are advanced in age and not many efficacious treatments are available. Therefore, early introduction of BSC including palliative radiotherapy is considered for the purpose of symptomatic relief and QOL improvement [133].

## Chapter 3: Treatment strategies for vaginal cancer

### General consideration

Patients suffering from vaginal cancer are elderly, and selected therapy is usually radiation therapy and CCRT, as is the practice with cervical cancer. The most common sites for vaginal cancer are the upper third of the vagina (56%), followed by the lower third (31%) and the middle third (13%) [134]. It is believed that the lymphatic routes of tumors localized in the upper two-thirds of the vagina drain mainly into the pelvic lymph nodes, while those in the lower third of the vagina drain into the inguinal lymph nodes. Therefore, the routes of metastasis vary according to the site and range of the primary tumor. Planning the treatment for vaginal lesions and for regional lymph nodes requires in consideration of the way of spread [135].

The type of surgical therapy is also chosen based on the site and range of occurrence of the primary lesion. At this time the physician must consider both removal of the primary lesion and regional lymph nodes simultaneously.

**Fig. 4:**
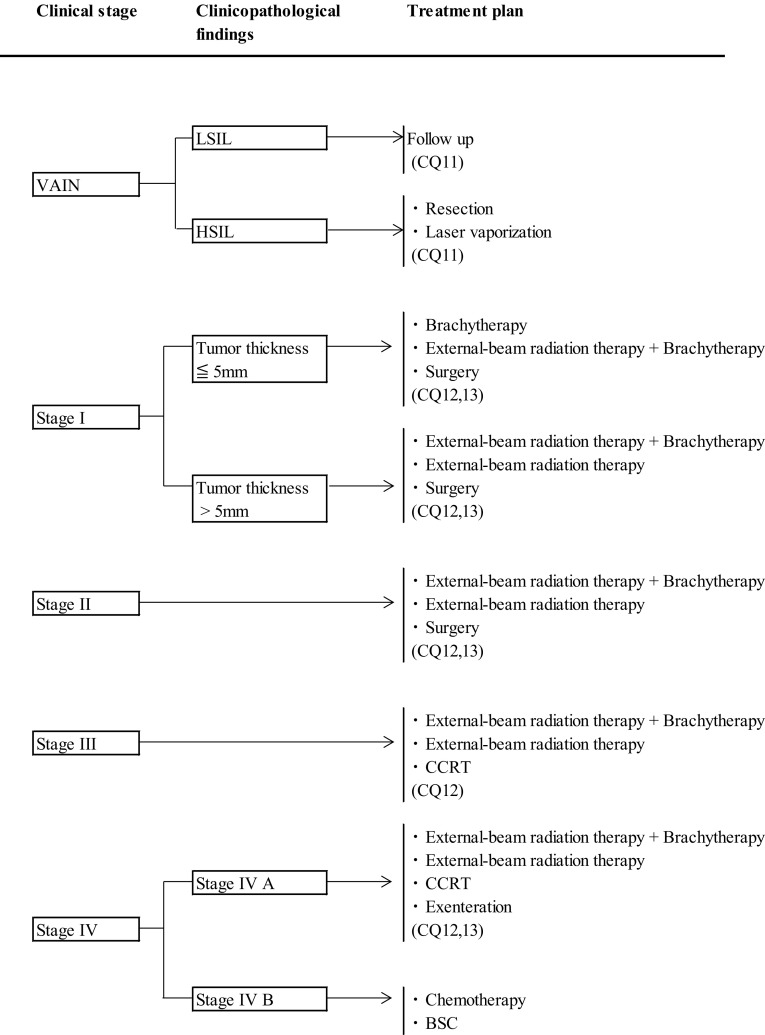
Primary treatment for vaginal cancer

Vaginal cancer is sometimes treated with chemotherapy in advanced or relapse cases, but evidence of its efficacy is scarce due to the small number of cases. Currently, it is used in conformity with the practice for cervical cancer (Fig. 4)

(1) Histopathological approaches

Most cancers originating in the vagina are squamous cell carcinomas. Histologically these can be divided into five types—keratinizing, non-keratinizing, basaloid, verrucous and warty. HPV is detected in about 80% of cases of vaginal squamous cell carcinoma, and HPV is most frequently detected in the non-keratinizing, basaloid and warty forms, in that order [136].

Vaginal intraepithelial neoplasia (VAIN) is a proliferation of atypical squamous cells within the vaginal epithelium but is not accompanied by interstitial infiltrate. As with cervical intraepithelial neoplasia (CIN), it is classified into three grades (VAIN 1, VAIN 2, VAIN 3) according to the level of atypical cell proliferation. Most cases of VAIN are caused by HPV infection [137]. In recent years, as with VIN, VAINs caused by HPV are classified into LSIL and HSIL [138, 139]. In these classifications, LSIL corresponds to VAIN 1 and HSIL corresponds to VAIN 2 and 3. The potential for malignancy is less clear for VAIN than for CIN and VIN (CQ11).

Although primary adenocarcinoma of the vagina is rare, clear-cell carcinoma in the women who are exposed to synthetic non-steroid estrogens such as diethylstilbestrol (DES) in utero has been reported [140]. Even among women never exposed to DES, the most common form of adenocarcinoma occurring in the vagina is clear-cell adenocarcinoma. Developmental abnormalities of the urogenital system, vaginal adenosis, and endometriosis are possible origins of the vaginal adenocarcinoma.

(2) Radiation therapy

Radiation therapy is a generally used treatment for vaginal cancer to preserve the function of adjacent organs (CQ12). However, because of the rarity of these tumors, no randomized controlled trials exist, and only retrospective studies in single facilities are reported. According to these reports, the significant prognostic factors are clinical stage and tumor size [141–151], and the main recurrence pattern is local recurrence [141, 143]. The 5-year pelvic control rate for radiation therapy is 80–90% in stage I, 50–70% in stage II, 50–60% in stage III and 30% in stage IV [141–143, 152]. The 5-year disease-specific survival rate is 80–90% in stage I, 70–80% in stage II, 50–60% in stage III and 10–20% in stage IV [142–144, 148], and the 5-year overall survival rate is 70–80% in stage I, 50–70% in stage II, 30–50% in stage III and 0–20% in stage IV [144, 149]. The 5-year rate of severe radiation morbidity is reported to be approximately 5–20% including ulceration of the vaginal mucosa and vulva, vaginal stenosis, rectal stenosis, rectovaginal fistula and fistula of the urinary bladder [141, 143–146, 148].

Methods of radiation therapy have recently changed in many ways. Brachytherapy has changed from low-dose-rate to high-dose-rate irradiation. Recently, with the growing adoption of image-guided brachytherapy (IMBT), it has become possible to evaluate doses accurately based on 3D treatment planning using CT or MRI [150, 151]. In external beam irradiation, 3D-CRT has become standard, and increasing numbers of facilities are using IMRT, improving dose distribution [152].

(3) Surgical therapy

In principle, the mainstay therapy for vaginal cancer is radiation therapy. However, surgical therapy is also an option, depending on the location and range of the focus/foci of disease (CQ13). In particular, in the case of vaginal cancer occurring in the upper third of the vagina, surgical therapy consisting of hysterectomy extended to the vagina is a good option [153]. If vaginal cancer is accompanied by widespread intraepithelial neoplasia in the vagina, a total vaginectomy may sometimes be selected. Moreover, in carefully selected cases of locally advanced vaginal cancer with no metastasis, pelvic exenteration is sometimes performed [154].

### Clinical questions and recommendations

#### Treatment for VAIN

CQ 11: What are the recommended treatments for VAIN?

Objective

The objective is to examine therapies for treating VAIN.

Recommendations

(1) Conduct periodic follow-up for LSIL (Grade A).

(2) Surgical therapies may include local, partial or total vaginectomy, depending on the case for HSIL. Laser vaporization may be considered for more conservative therapy (Grade C1).

(3) Loop electrosurgical excision procedure (LEEP) is not recommended due to the risk of injury to the urinary bladder or rectum (Grade D).

Comments

Many reports concerning VAIN have been published in accordance with the categories VAIN 1 to VAIN 3, advocated in the WHO classification of 2003 (3rd edition). The frequency of occurrence of VAIN is low, approximately 1% of that of CIN, which is also caused by HPV infection [155]. Many VAIN cases are combined with VIN and CIN as part of the effects of HPV infection [156, 157]. VAIN is often seen in patients whose immune systems are compromised, whether from an immunodeficiency disease such as human immunodeficiency virus (HIV) or long-term use of immunosuppressants or steroids in connection with a transplant procedure [158]. In addition, VAIN cases are often associated with a past hysterectomy due to CIN or cervical cancer, or a history of radiation therapy [155, 159–162]. Like VIN, VAIN can present with multiple foci of disease, or with a focus of disease spread over a wide area. Before initiating treatment, the entire vagina must be examined thoroughly.

In a retrospective examination of therapeutic intervention in cases of VAIN, treatment was provided in 30% of cases of VAIN 1, 77% of VAIN 2 and 93% of VAIN 3 [163]. In LSIL, which corresponds to VAIN 1, spontaneous regression of disease occurs in roughly half of all cases with periodic follow-up alone. Accordingly, proactive treatment is often reserved for HSIL, which corresponds to VAIN 2 and VAIN3 [157, 159]. As reports indicate that latent cancers are hidden in 12–13% of HSIL cases [160, 164], surgical excision may be considered in cases with suspicion of invasive carcinoma. Because >80% of VAIN cases occur in the upper third of the vagina [160, 164], treatment with partial vaginectomy is often reported [159, 164]. Depending on the range and sites of the foci of disease, local excision or total vaginectomy may be performed [160, 162, 165]. A variety of excision approaches are also adopted, including transvaginal, laparotomic and laparoscopic surgery, and these may or may not be accompanied by hysterectomy [162, 166]. Because of the anatomical structure of the vagina, complications such as damage to the urinary bladder and rectum and fistulation have been reported after surgical excision [165, 167]. Moreover, VAIN patients sometimes have a history of hysterectomy or radiation therapy; these conditions can render surgical excision difficult. Physicians must consider individually when selecting a treatment method.

When invasive carcinoma can be ruled out after sufficient examination, the most widely used preservative treatment is laser vaporization [167–170]. Unfortunately, recurrence rates are high in these cases, requiring repeated treatment or surgical excision, and continuous observation [162, 163]. The use of LEEP is reported [171], but is not recommended due to the high risk of damage to the urinary bladder and rectum [172]. In cases that are resistant to treatment, intracavitary irradiation [173, 174] and local administration of 5-fluorouracil [175] have been reported. As with surgical excision, the risk of impairment of the urinary bladder and rectum is considerable.

Cases of VAIN are few and are often accompanied by primary illnesses that compromise immunity, combined with VIN or CIN, or are associated with a history of hysterectomy or radiation therapy. These circumstances render it difficult to establish clinical trials to gather evidence, such as randomized controlled trials. Cases of VAIN need to be managed on an individual basis, accompanied by long-term periodic follow-up.

#### Radiation therapy for the vaginal cancer

CQ 12: How should radiation therapy be applied, and what methods should be used?

Objective

The objective is to examine how to apply radiation therapy to vaginal cancer and the methods of radiation therapy to use.

Recommendations

(1) Brachytherapy may be performed alone or in combination with external beam irradiation for stage I vaginal cancer with tumor thickness of ≤5 mm (Grade C1).

(2) External beam irradiation may be performed in combination with brachytherapy or alone for stage I vaginal cancer with tumor thickness >5 mm, or for vaginal cancer in stages II to IVA (Grade C1).

(3) Concurrent chemotherapy with a single platinum-based drug or combination of this drug may be considered (Grade C1).

Comments

Radiation therapy to treat vaginal cancer may consist of brachytherapy, external beam irradiation, or a combination of the two, depending on the condition of the focus of disease.

The JASTRO guidelines state that brachytherapy can be applied as intracavitary irradiation alone in cases of stage I vaginal cancer in which the tumor is ≤5 mm thick [151, 176]. However, the intra-pelvic recurrence rate of patients treated with intracavitary irradiation alone for stage I vaginal cancer was 20–33% [143, 146]. Physicians must take care regarding underestimation of invasion of the submucosa. If there is suspicion of invasion to the submucosa, intracavitary irradiation must be combined with external beam irradiation regardless of the size of the tumor even for stage I vaginal cancer. External beam irradiation is performed in combination with brachytherapy or alone for vaginal cancers >5 mm in thickness, or for vaginal cancer in stages II to IVA.

The external beam irradiation is applied first. If the primary lesion is in the upper two-thirds of the vagina, the CTV includes the area of the obturator lymph nodes, internal and external iliac lymph nodes, common iliac lymph nodes and presacral lymph nodes. If the primary lesion is in the lower third of the vagina, the CTV covers the groin [141, 143]. The CTV must include the area of the rectal lymphatic nodes if the tumor invades the posterior wall of the vagina, and must include the vulva if it invades the vaginal entrance. Irradiation with central shielding should begin at 30–40 Gy and rise to 45–50 Gy [143]. After central shielding, additional doses are added via brachytherapy to the primary lesion. When the dose reaches 30–40 Gy, the additional dose is administered by brachytherapy if the boundaries of the residual tumor are clear, invasion around the vagina is ≤50–60%, and no deep invasion of the paracolpium is observed [3]. A cylindrical or ovoid applicator may be used to conduct intracavitary irradiation if the focus of disease is localized in the vaginal fornix, or if the tumor is ≤5 mm thick. According to the consensus guidelines of the American Brachytherapy Society (ABS) on interstitial irradiation of vaginal cancer, however, interstitial irradiation should be considered if the tumor is >5 mm in thickness [150]. In the past, brachytherapy typically consisted of low-dose irradiation, but today most institutions have replaced this practice with high-dose irradiation. According to a retrospective study of radiation therapy of vaginal cancer using high-dose irradiation, the treatment results, recurrence patterns and adverse events are all similar to those of low-dose irradiation in the past [149, 177–180]. Current evidence does not permit the recommendation of particular points for evaluating the dose in brachytherapy or for selecting the method of fractionation or dose. However, the typical regimen is three to six treatments with a dose of 4–7 Gy per treatment, taking the entire vaginal mucosa as the CTV [150, 151]. External beam irradiation to the clinical focus of disease with a dose of 65–70 Gy alone may be performed in the following cases—where the tumor has penetrated deeply and the boundary is unclear, or if shrinking of the tumor using initial external beam irradiation is insufficient and the physician judges that the tumor cannot be treated with the appropriate dose using brachytherapy, or if the facility is not able to perform brachytherapy [141, 152].

In recent years, CCRT has been used in radiation therapy for locally advanced vaginal cancer, following the evidence of cervical cancer. Several reports examined the efficacy of CCRT using drugs such as cisplatin and 5-fluorouracil to improve the tumor control rate in locally advanced vaginal cancer, but these are only retrospective studies in single institutions, with small sample sizes [143, 181–183]. Because of the rarity of this disease, it is difficult to test the efficacy of concurrent chemotherapy through randomized controlled trials. For this reason, it is judged appropriate to apply the results of clinical trials regarding cervical cancer, based on the similarities of organ site, causal factors and histopathology. Physicians must consider concurrent use of chemotherapy in combination with radiation therapy if the tumor is stage III or IVA, >4 cm in diameter, or positive for lymph node metastasis, and general health is good and organ function is preserved [143, 184]. A pattern of care analysis in the United States determined that the use of CCRT with cisplatin as the main drug has been increasing since 1999 [185]. Nonetheless, due caution is in order regarding adverse events, given that patient age is typically higher for vaginal cancer than for cervical cancer [181].

#### Surgical treatment for invasive vaginal cancer

CQ 13: How should surgical therapy be applied, and what methods should be used?

Recommendations

(1) Surgical therapy may be considered in clinical stages I and II if the tumor is located in the upper third of the vagina (Grade C1).

(2) Surgical therapies may include radical hysterectomy, or modified radical hysterectomy and pelvic lymphadenectomy, and vaginectomy with sufficient excision margin (Grade C1).

(3) Pelvic exenteration may also be considered in clinical stage IVA disease (Grade C1).

Comments

In 56% of vaginal cancers the tumor is in the upper third of the vagina and about half of those are located in the posterior wall. If a tumor extends to the middle or lower third of the vagina, it will probably require pelvic exenteration or vulvectomy to remove it completely. Because of the significant reduction in QOL with such an invasive procedure, physicians tend to select radiation therapy. In the case of a stage I or II tumor in the upper vagina, a radical or modified radical hysterectomy is often selected, in combination with vaginectomy with sufficient margin, as is the practice with cervical cancer.

In a retrospective examination of 100 cases of primary vaginal cancer at a single institution, it was reported that the prognosis was better for surgical therapy than for radiation therapy alone if the tumor was located in the upper one-third of the vagina in clinical stage I or II [186]. A report from Japan analyzed 51 cases of vaginal cancer and found a similar trend [147]. These reports deal only with stage I and II cases in which the tumor extends no further than the upper third of the vagina. If the tumor is larger, extending beyond the upper third, radiation therapy is selected. In the case of small tumors localized in the lower vagina, however, surgical therapy may be considered. In an analysis of 4885 cases of primary vaginal cancer conducted by the US National Cancer Database, the 5-year survival rate was found to be 90% for surgical therapy and 63% for radiation therapy in stage I; in stage II, the figures were 70 and 57%, respectively. These figures indicate a much better trend for surgical therapy than for radiation therapy with probable selection bias [136, 187].

In histological terms, the majority (79–85%) of vaginal cancers are squamous carcinomas, while 6–14% are adenocarcinomas [134, 136]. When the type of therapy is selected by histological category, stage I and II adenocarcinoma (especially clear-cell adenocarcinoma) is poorly sensitive to radiation therapy, and surgical therapy is recommended [134, 188].

Surgical therapy may consist of vaginectomy preserving a sufficient excision margin, radical hysterectomy and pelvic lymphadenectomy. Curative surgery for a tumor in the lower third of the vagina must be combined with inguinal lymphadenectomy. Postoperatively, if risk factors such as a positive stump or lymph node metastasis are present, radiation therapy is recommended as an adjuvant therapy [147, 154, 188].

When vaginal cancer is discovered after a hysterectomy, surgical therapy may also be considered if the tumor is localized in the vaginal wall. Partial vaginectomy is conducted for carcinoma in situ. For invasive carcinoma in the upper third of the vagina, a vaginectomy including paracolpium, combined with a pelvic lymphadenectomy is recommended [188].

Extended surgery such as pelvic exenteration may be considered if the patient has the invasive tumor to the rectum or urinary bladder, a rectovaginal or vesicovaginal fistula, or local recurrent tumors after radiation therapy [134, 188]. According to a report by the US National Cancer Database, the 5-year survival rate in stage III and IV is 47% for surgical therapy alone and 35% for radiation therapy alone. However, the 5-year survival rate rises to 60% when surgical and radiation therapy are combined, indicating a favorable trend when surgery is added [136]. There should be selection bias not to perform surgery for cases with tumors extending to the pelvic wall, and to select radiation therapy for highly invasive cases. Consequently, surgery may be performed only for cases where the prognosis tends to be better.

#### Follow-up of vaginal cancer

CQ 14: How should periodic follow-up be handled after treatment?

Objective

The objective is to examine appropriate intervals and methods for periodic follow-up to deal with recurrences and complications associated with treatment.

Recommendations

(1) A rough guide to the intervals for periodic follow-up after treatment is as follows (Grade C1):

First and second years: once every 1–3 months

Third to fifth years: once every 6 months

Sixth and subsequent years: once a year

(2) Conduct medical interviews, inspection, palpation, cytology, biopsy, chest X-ray examination, tumor markers and CT (Grade C1).

Comments

No evidence exists to suggest that diagnosis for recurrence through periodic follow-up after treatment for vaginal cancer leads to improved prognosis. No reliable research has been carried out on the intervals and methods of examination for periodic follow-up, and no consensus on these matters has been reached.

The guidelines of the NCI of the United States include no recommended interval for periodic follow-up, except that cytodiagnosis and image examination should be conducted when physical findings lead the physician to suspect recurrence, or when the patient reports subjective symptoms [189].

According to the American College of Radiology (ACR) Appropriateness Criteria, periodic follow-up is recommended once every 3 months for the first 2 years after treatment, followed by periodic observation at longer intervals thereafter. However, the grounds for these recommendations are not explained [190]. Some retrospective studies of multiple cases showed 70–80% of relapsing cases occur within the first 2 years after initial treatment. The recurrence rate declines for the third and subsequent years, but recurrences have been confirmed even after 5 years [141, 142]. While the factor most highly correlated with recurrence is stage [143, 148], localized recurrence is the most common pattern regardless of stage [141–143, 148]. Recurrence in the lymph nodes is also common, with metastasis frequently occurring in the pelvic nodes, and in the inguinal nodes when the primary site is in the lower one-third of the vagina [141]. According to a report on 301 cases of vaginal cancer that were treated with radiation therapy, recurrence was localized in the vagina in 69 cases (23%), occurred in the pelvic nodes in 21 cases (7%) and the inguinal nodes in 12 cases (4%), while distant metastasis occurred in 44 cases (15%). Prognosis after recurrence was extremely poor, with a 5-year survival rate of only 20% even for local recurrence. The rate dropped to 4%, indicating that recovery is extremely difficult after recurrence in cases of lymph node recurrence or of distant metastasis [141].

Medical interviews, inspection and palpation are vital components of medical examinations in periodic follow-up after treatment. Recurrence can be confirmed using cytodiagnosis and biopsy if recurrence is suspected in the vagina or superficial lymph nodes. Other examination items useful in finding recurrences in other locations include chest X-ray, tumor markers, CT, MRI and FDG-PET. However, no standard examinations or schedules have been established.

The current first choice for treatment, radiation therapy, is associated with long-term complications. The rate of occurrence of Grade 3–4 adverse events after radiation therapy is reported as 13–17%, and can occur even 10 years after treatment [141, 143]. Frequent complications include radiation proctitis, ileus, homorrhagic cystitis, rectovaginal fistula, vesicovaginal fistula, gastrointestinal obstruction and urethral stenosis [141, 143]. Because such complications can severely damage a patient’s QOL, long-term periodic follow-up to check for these conditions is vital.

## Chapter 4: Treatment strategies for other vulvar and vaginal cancers

### General consideration

Malignant diseases occurring in the vulva other than squamous carcinoma include cancers and sarcomas originating in the skin, such as Paget’s disease and associated invasive carcinomas, malignant melanomas, cancers originating in the Bartholin’s glands, and basal-cell carcinomas.

Malignant diseases occurring in the vagina other than squamous carcinoma include adenocarcinoma, malignant melanomas and sarcomas. Because Paget’s disease and malignant melanomas essentially originate in the skin, detailed guidelines have already been published for their general treatment as diseases of the skin [191]. However, gynecologists encounter these two diseases relatively commonly as lesions of the vulva and vagina, and conditions peculiar to the vulva and vagina must be taken into account in their treatment. Standard treatment methods for other tumors in the vulva and vagina are not established due to their rarity. Physicians must deal with these diseases on a case-by-case basis according to the characteristics of each tumor and the condition of each patient.

(1) Histopathological approaches

Vulvar Paget’s disease is characterized by intraepithelial proliferation of large atypical cells other than the squamous epithelium and melanocytes. This disease can be divided into two types—primary Paget’s disease, which originates in the skin of the vulva; and secondary Paget’s disease, which originates from malignancies in surrounding organs such as the rectum, anal canal, urinary tract and uterine cervix. Primary Paget’s disease can be further divided into tumors of intraepidermal origin and those originating from cancer of skin appendages or the Bartholin’s glands [192]. In secondary Paget’s disease, treatment including the primary organ must be considered. Histologically, Paget’s cells are larger than the surrounding epithelial cells and exist as isolated cells or nests with various sizes. Occasionally Paget’s cells appear in tubular form or as signet-ring cells. Primary and secondary Paget’s disease can be distinguished immunohistochemically. The tumor cells of primary Paget’s disease are positive for cytokeratin (CK) 7 and GCDFP-15 but generally negative for CK20, while the cells of rectal and anal canal carcinomas are CK7-negative, CK20-positive and GCDFP-15-negative. Urothelial carcinoma cells are both CK7- and CK20-positive, and GCDFP-15-negative [193, 194].

Malignant melanomas are malignant tumors of melanocytes. Malignant melanomas originating in the vulva and vagina are histologically classified into three categories—mucosal, superficial-spreading and nodular. In the vulva, the mucosal and superficial-spreading types are dominant, while the nodular type is common in the vagina [195, 196]. In histological diagnosis, positive result in immunostaining for S-100 protein, HMB-45, and melan-A support the confirmation of melanocytic features. Stage is determined by the staging system of cutaneous melanomas [197].

(2) Surgical therapy

Paget’s disease

Vulvar Paget’s disease presents in several forms as previously mentioned in histological approaches. Physicians need to be thoroughly familiar with these various types of Paget’s disease when providing treatment (CQ15).

The first choice in treatment of vulvar Paget’s disease without invasive carcinoma is wide local excision in due consideration of the excision range. The local relapse rate after excision is high (32–37%) [198, 199] for at least three reasons. First, this disease tends to present with multiple foci of disease. Second, foci of disease are sometimes present at sites that appear normal at the gross (visible) level. Third, the boundaries of tumors can be difficult to distinguish in the vulva, due to the region’s natural pigmentation as well as conditions such as eczema and inflammation. Fortunately, while the high relapse rate often requires repeated treatment, intraepithelial lesions transition gently, and prognosis tends to be exceptionally favorable [198–201]. Some researchers variously advocate performing a biopsy (mapping biopsy) on areas around the focus of disease that appear normal preoperatively [202] or performing a frozen section examination during surgery [203, 204], to confirm that no focus of disease remains in the excision stump. However, other reports argue that these procedures are not necessarily effective [205, 206].

Surgery is performed in accordance with procedures for invasive vulvar cancer if Paget’s disease is accompanied by invasive carcinoma. The vulva is the most common site (82%) for invasive extramammary Paget’s disease in women according to a report on a cohort study [207]. In 1439 cases of invasive extramammary Paget’s disease, the disease-specific 5-year survival rate was 95% in cases with localized lesion, 85% for lesions advanced to adjacent organs and 53% for distant metastasis. The prognosis was reported to be significantly better for the surgery group compared to the non-surgical group.

Malignant melanomas

Vulvar and vaginal malignant melanomas are mucosal. They are distinct from cutaneous malignant melanomas, which comprise >98% of malignant melanomas. Cutaneous malignant melanomas have been treated by surgical therapy and pharmacotherapy, based on their clinical and histopathological characteristics. Similarly, thorough surgical excision is a principle for malignant melanomas of the vulva and vagina, but restrictions exist concerning anatomical difficulties and postoperative QOL. Currently, pharmacotherapy is also used, following the practice for cutaneous malignant melanomas (CQ16).

A margin of ≥2 cm of normal tissue is generally mandatory in excision of vulvar malignant melanomas, but if tumor infiltration is ≤1 mm, a margin of ≥1 cm is regarded sufficient [208–210]. Radical vulvectomy is not necessarily required, and radical local excision may be applicable, as is the case with squamous carcinoma. Even in such cases, the subcutaneous adipose tissue should be totally resected just above the fascia. Conversely, in cases with more tumor infiltration and/or spread, wider excision is usually performed. Inguinal lymph node metastasis is a definite poor prognostic factor for vulvar malignant melanomas. Lymphadenectomy (biopsy) is required to confirm the presence of metastasis, but as the therapeutic significance of this procedure is obscure [211], SLN biopsy has recently been applied to avoid systemic node dissection. This approach is proclaimed to have >85% negative predictive value, but the procedure remains at the research stage and should be performed only by highly experienced teams [212, 213].

Most vaginal malignant melanomas are diagnosed at an advanced stage, rendering prognoses extremely poor. Extended surgeries such as radical vaginectomy, pelvic exenteration, and inguinal and pelvic lymphadenectomy have been performed, but the significance of these procedures is not conclusive [214, 215]. In small localized cases with shallow infiltration, local excision with sufficient margin followed by postoperative treatment may provide favorable outcomes [216].

Other vulvar cancers

For other vulvar cancers, the most commonly chosen treatment option is surgery, but the procedure needs to be determined based on the clinical characteristics. For example, as tumors originating in Bartholin’s glands tend to infiltrate deeper than ordinary vulvar cancers, excision must extend deeper in order for curative surgery to be effective [217, 218]. Because the excision stumps have a high positive rate for foci of disease, addition of preoperative and postoperative radiation therapy has been reported, but prognosis is still poor [218, 219]. For basal-cell carcinoma, local excision is usually judged sufficient because metastasis is rare. However, a local relapse rate of 10–22% is reported, and caution is in order [220].

(3) Radiation therapy

In vulvar Paget’s disease, radiation therapy is applied in elderly patients or patients with medical complications rendering the condition inoperable, or in advanced disease rendering surgery inappropriate, and in cases of postoperative recurrence. The only reports on the effectiveness of this therapy are retrospective studies of a small number of cases and case reports. The study with the largest number of cases was reported from Japan, consisting of 22 cases of radiation therapy for external genital Paget’s disease, including 12 cases of vulvar Paget’s disease. The treatment results consist of 10 cases of definitive radiation therapy, eight cases of postoperative irradiation and four cases of postoperative recurrence. The 5-year local control rate for these cases was 84%, the disease-specific survival rate was 73%, and the overall survival rate was 53%, with no serious radiation morbidity [221].

Definitive radiation therapy is rarely the first choice for malignant melanomas as their sensitivity to irradiation is low. However, heavy-particle radiation therapy has been conducted on malignant melanomas on a trial basis in the gynecology field, as it has a stronger biological effect than conventional radiation therapy. Some effectiveness has been reported [222].

As an adjuvant treatment to surgery, postoperative irradiation has been conducted in cases of lymph node metastasis, for which the risk of recurrence is high. This procedure has been shown to improve the control rate in the lymph node area [223, 224]. However, its contribution to improvement of prognosis has not been established, and problems of increasing late radiation morbidity have been pointed out. The decision on whether to apply these procedures must be judged on a case-by-case basis.

### Clinical questions and recommendations

#### Surgical treatment and alternative therapy for vulvar Paget’s disease

**Fig. 5:**
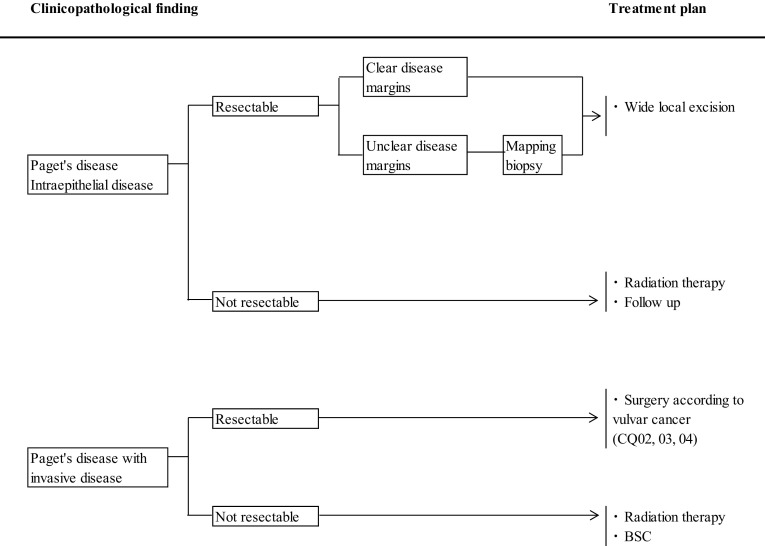
Primary vulvar Paget’s disease

CQ 15: What treatments are recommended for primary vulvar Paget’s disease? (Fig. 5)

Objective

The objective is to examine treatment methods for primary vulvar Paget’s disease.

Recommendations

(1) Wide local excision with sufficient excision margin is recommended for intraepithelial lesions unaccompanied by invasive carcinomas (Grade B).

(2) A mapping biopsy may be considered for lesions without a clear gross boundary (Grade C1).

(3) Surgery following the practice for ordinary invasive vulvar cancer is recommended for lesions accompanied by invasive carcinomas (Grade B).

(4) Radiation therapy may be considered in inoperable cases, or in cases of recurrence (Grade C1).

Comments

For intraepithelial vulvar Paget’s disease without invasive carcinoma, the recommended treatment is wide local excision, giving careful consideration to the excision range. The local recurrence rate after excision is reported to be as high as 32–37%. However, progression in intraepithelial Paget’s disease is gentle, and its prognosis is extremely favorable [198, 199].

No highly reliable evidence exists concerning the excision range (excision margin) required for the complete excision of vulvar Paget’s disease. According to the discussion of extramammary Paget’s disease in Treatment Guidelines of Malignant Skin Tumors, Japan, excision may be conducted with a margin of about 1 cm if the focus of disease has a clear gross boundary or the site is judged negative for metastasis in a mapping biopsy. If the gross boundary is not clear, an excision margin of about 3 cm is recommended [191]. These recommendations are based on the findings of two studies. First, according to data from mapping biopsies of extramammary Paget’s disease and from implementation of Mohs surgery (surgery in which the entire excision stump is confirmed during surgery using frozen sections), an excision margin of ≥3 cm is required for extramammary Paget’s disease. Second, in a study of 46 cases in which a margin of 1 cm was obtained for lesions with clear gross boundaries on ordinary skin, the difference between the gross boundary and the histological boundary was small [225, 226]. In a report on 33 cases of vulvar Paget’s disease in which frozen section diagnoses were conducted during surgery to determine excision range, the excision stump was positive in 44% of cases, while in cases where these intraoperative diagnoses were not conducted, the positive rate was 56% [199]. Another study, reporting on 30 cases of vulvar Paget’s disease, found that in nine cases where the excision range was decided on gross appearance, the positive rate for the excision stump was 67%, but in 18 cases where the excision range was decided based on mapping biopsies and intraoperative checks during surgery, the positive rate declined to just 39% [202]. Furthermore, in a single-facility retrospective study in Japan of 18 cases where the excision margin was determined so as to ensure a negative mapping biopsy, the stump was negative in postoperative histopathological diagnosis and no recurrences were confirmed [227]. These results testify that mapping biopsies and frozen section checks during surgery reduce the positive rate in excision stumps. However, other studies found that in cases where excision stumps were positive, the recurrence rate was 31–70%, and even when it was negative, recurrence was confirmed in 18–38% of cases, indicating that recurrence can happen regardless of the condition of excision stumps [197, 228, 229].

The depth of the excision is sufficient to extend through all dermal layers and slightly into the subcutaneous fat in cases of intraepithelial lesion because the tissue of the vulvar appendages of the skin is only ≤4 mm thick. For intraepithelial lesions without invasive carcinomas, inguinal lymphadenectomy should not be conducted, as lymph node metastasis is absent.

Because vulvar Paget’s disease can progress into adjacent structures such as the urethra, vagina and anus, it is necessary to confirm sufficiently that such progression has not occurred when setting the excision range. These mucous membrane adjacent organs must also be excised as far as possible if such progression is confirmed. Within the anus, excision is possible as far as the pectinate line.

Although the prognosis for intraepithelial vulvar Paget’s disease is favorable, postoperative local recurrence is frequent. According to a report on 17 cases of recurrence out of 28 initial surgery cases, one to three additional excisions were performed in 14 of the 17 recurrence cases, and the lesions were eliminated in 80% of these cases [229].

Whether for initial treatment or for recurrence treatment, surgical excision should be considered first. However, as patients are typically elderly, averaging 68–70 years of age at the time of diagnosis, the focus of disease is often wide. For this reason, a less invasive treatment has come to be adopted, in which photodynamic therapy (PDT), external use of imiquimod, or laser vaporization, are tried alone or in combination [230–232]. However, the reports on these treatment methods consist mainly of retrospective studies and case reports, and the observation period is often brief. No randomized controlled trials have been performed to compare the recurrence rate and survival rates of these conservative treatments to those of surgical excision. At present, it is not possible to recommend these procedures for daily practice.

When intraepithelial Paget’s disease is combined with invasive carcinoma, surgery is performed as for normal invasive vulvar cancer. Recommended therapies include local excision with securing an excision margin of ≥1 cm, or radical vulvectomy, with inguinal lymphadenectomy. No data exist to suggest that inguinal lymphadenectomy improves survival rate if the condition is combined with invasive carcinoma. However, a report on 76 cases of vulvar Paget’s disease including 22 cases accompanied by invasive carcinoma reported that biopsy or excision of the inguinal lymph nodes was conducted in 19 cases and metastasis to the inguinal lymph nodes was confirmed in nine of these cases, six of which resulted in death [200]. Because the inguinal lymph nodes are a common site for metastasis, and lymph node metastasis is an important prognostic factor, dissection should be considered in cases with underlying invasive cancer.

No standard treatment method is established for invasive vulvar Paget’s disease with metastasis, or recurrent disease. No randomized controlled trials or prospective trials have been reported regarding the use of postoperative radiation therapy in cases of vulvar Paget’s disease combined with invasive carcinoma or metastasis to the inguinal lymph nodes, and the usefulness of the treatment is difficult to assess at this point. Similarly, no regimen of chemotherapy is suitable for recommendation in the case of advanced vulvar Paget’s disease with distant metastasis. A small number of reports exist for single-drug or multi-drug chemotherapy following the practice for breast cancer or gastrointestinal cancer. However, the response rate and survival benefit still remain unclear.

If surgery is impossible due to advanced age, medical complications or progressive disease, or if postoperative recurrence occurs and no other treatments are available, radiation therapy may be considered. One report from Japan details the results of radiation therapy in 22 cases of Paget’s disease in the external genitalia as previously cited [221]. X-rays or electron beams were used for radiation therapy, and effort was made to irradiate the entire focus of disease with a uniform dose. If invasion is confirmed from the dermis to the hypodermis, the danger of lymph node metastasis exists, and preventive irradiation of the lymph node area may be considered [233]. Although no standard dose fractionation is established, the required total dose is believed to be 50 Gy for non-invasive cases and 55–65 Gy for invasive cases and cases combined with invasive adenocarcinoma, with 1.8–2 Gy fraction [221, 233]. For very elderly patients, periodic follow-up alone is another option in vulvar Paget’s disease not accompanied by invasive carcinoma.

#### Treatment of malignant melanoma of the vulva and vagina

CQ 16: What treatments are recommended for malignant melanomas?

Objective

The objective is to exhibit appropriate treatments for vulvar and vaginal malignant melanomas.

Recommendations

(1) Excision of the primary lesion is recommended if distant metastasis is not confirmed. (Grade B).

(2) SLN biopsy with cooperation of dermatologists may be useful for determining the disease stage (Grade C1).

(3) Dacarbazine-based chemotherapy may be considered. (Grade C1).

Comments

The tumor thickness (TT) of the primary lesion is the most significant prognostic factor for vulvar malignant melanomas [211]. Because the TT is correlated with degree of progression of the tumor, the excision range should be decided in a two-step manner after evaluating the TT. Namely, excisional biopsy is initially performed with entire layers of subcutaneous adipose tissue above the basal fascia taking a 2-mm margin around the primary lesion [191]. A partial resection is acceptable if the lesion is too large or too close to the urethral opening to suture the wound defect since difference in prognostic outcome exists although the diagnostic accuracy is decreased [191]. Based on tumor histology and TT, the additional excision range is decided as follows —the margin from the tumor border should be set at 3–5 mm, 1 cm, and 2 cm for in situ lesions, TT ≤ 2 mm, and TT > 2 mm, respectively [191, 234]. As survival prognosis cannot be improved even with wider excision [195, 235], the excision range should be addressed near to the urethral opening and anus with careful consideration of postoperative QOL. The prognostic outcome is not worsened by the interval between the primary excision and the second excision, and is superior in two-step excision after confirming the TT to one-step wide-area excision [191].

For vaginal malignant melanomas, curative excision is also preferred; however, a pelvic exenteration is frequently necessary due to the tumor location and multiple foci. Because no difference in prognosis exists between highly radical surgery and local excision, a combination of local excision with irradiation would be preferred both for less complications and better local control and survival [236].

In vulvar malignant melanomas, those with regional lymph node metastasis exhibit poor prognostic outcome, but lymph node metastasis does not in itself comprise an independent prognostic factor [211]. The significance of systematic lymphadenectomy is low as it did not provide better prognostic outcome even with radical vulvectomy compared to wide local excision alone [237, 238]. Curative lymphadnectomy may be considered to provide long-term survival for young patients who bear only a couple of lymph node metastasis without extracapsular invasion [191]. As for vaginal malignant melanomas which are accompanied with lymph node metastasis even at early stages, the significance of systematic lymphadenectomy on prognosis is still unclear.

SLN biopsy is so far a case-trial procedure for vulvar and vaginal malignant melanomas [239]. As an SLN biospy is expected to detect and excise microscopic metastasis, it is already recommended for those bearing cutaneous malignant melanomas with 1–4 mm thickness due to its safety and efficacy [191]. A phase III trial also exhibited that a 10-year disease-free survival rate for those with TT >1.2 mm was significantly higher if an SLN biopsy was performed [240]. Thus, the SLN biopsy, supervised by a certified dermatologist with experience in treating malignant skin tumors, would be reasonably applied to the treatment for vulvar and vaginal malignant melanomas.

Postoperative adjuvant treatment is widely performed for vulvar and vaginal malignant melanomas although its efficacy has not been well clarified. DAV-Feron therapy is a three-drug combination chemotherapy of dacarbazine (DTIC), nimustine and vincristine combined with a local injection of interferon-beta, and it has been employed in Japan as a prognosis-improving regimen for high-risk cutaneous malignant melanomas [241]. Postoperative immunotherapy (interferon-alpha and interleukin) can reduce recurrence but its survival benefit is uncertain [191]. In applying these regimens on vulvar and vaginal malignant melanomas, supervision by well-experienced dermatologists is mandatory.

The efficacy of postoperative irradiation to prevent node relapse of cutaneous malignant melanoma is demonstrated not only in retrospective studies [223] but a randomized controlled trial (ANZMTG 01. 02/TROG 02. 01) [242]. However, whether to conduct irradiation or not should be determined for each case by evaluating the risk of recurrence since postoperative irradiation is highly accompanied with adverse events such as lymphedema and because its contribution to better survival is not clear. Hypofractionation using brief exposure of high doses per treatment is expected to be safe and effective against malignant melanomas, although there is no standard regimen established to date.

The prognostic outcome of patients bearing distant metastasis is so poor that extensive excision should be avoided. Excision of metastasis is preferred only for those bearing a solitary tumor with good performance status. Irradiation to metastases in bones or central nerves relieves symptoms in half of cases, and stereotactic irradiation reduces tumor growth by 90% even for multiple brain metastases without active foci other than the brain [191].

Systemic chemotherapy using DTIC is applied on those bearing metastasis, but the response rate is only 20% and the long-term CR rate is <2% [191]. The initial response rate of DTIC is higher with other anti-cancer drugs compared to DTIC alone. However, there is no randomized trial which demonstrates the superiority of survival period of DTIC-combination therapy to DTIC alone.

For advanced malignant melanomas, several systemic immunotherapies have been investigated—adoptive cellular immunotherapy, cancer vaccines, and cytokine treatments such as interferon-alpha and interleukin-2. The response rates of these treatments, however, remain at most 10% [191]. Recently, much attention has been paid to immune-checkpoint targeting drugs (anti-CTLA-4 antibodies and anti-PD-1 antibodies) [243, 244] and to molecular-targeting drugs for tumor-specific genetic mutations. An anti-PD-1 antibody, nivolumab, and an anti-CTLA-4 antibody, ipilimumab, would deliver longer survival to cutaneous malignant melanomas, and other immune-checkpoint targeting drugs used overseas are also expected to be available in the near future. However, at present it is well demarcated that pharmacotherapies are tailored to mucosal-type vulvar and vaginal malignant melanomas, in which no standard treatments have been established. A multi-institutional prospective study should be conducted to assess the efficacy of these drugs on rare mucosal malignant melanomas including vulvar and vaginal malignant melanomas.

## Electronic supplementary material

Below is the link to the electronic supplementary material.

Supplementary material 1 (PDF 679 kb)

Supplementary material 2 (XLSX 19 kb)

## Abbreviations

ABS: American Brachytherapy Society
ACR: American College of Radiology
BP: Bowenoid papulosis
BSC: Best supportive care
CCRT: Concurrent chemoradiotherapy
CIN: Cervical intraepithelial neoplasia
CQ: Clinical question
CR: Complete response
CT: Computed tomography
CTV: Clinical target volume
DES: Diethylstilbestrol
DTIC: Dacarbazine
EORTC: European Organization for Research and Treatment of Cancer
FDG-PET: 2-Deoxy-2-[18F] fluoro-d-deoxy glucose-positron emission tomography
FIGO: International Federation of Gynecology and Obstetrics
GOG: Gynecologic Oncology Group
HIV: Human immunodeficiency virus
HPV: Human papillomavirus
HSIL: High-grade squamous intraepithelial lesion
IGBT: Image-guided brachytherapy
IMRT: Intensity-modulated radiation therapy
ISSVD: International Society for the Study of Vulvovaginal Disease
JGOG: Japanese Gynecologic Oncology Group
LEEP: Loop electrosurgical excision procedure
LSIL: Low-grade squamous intraepithelial lesion
MRI: Magnetic resonance imaging
NCI: National Cancer Institute
PDT: Photodynamic therapy
PR: Partial response
PS: Performance status
QOL: Quality of life
SIL: Squamous intraepithelial lesion
SLN: Sentinel lymph node
VAIN: Vaginal intraepithelial neoplasia
u/d VIN: Usual/differentiated vulvar intraepithelial neoplasia
WHO: World Health Organization
3D-CRT: Three-dimensional conformal radiation therapy
