# Supplementary material for: Japan Society of Gynecologic Oncology guidelines 2015 for the treatment of vulvar cancer and vaginal cancer
Source: Int J Clin Oncol. 2017 Nov 20;23(2):201–34. doi: 10.1007/s10147-017-1193-z (PMC5882649; doi:10.1007/s10147-017-1193-z)
Supplement: Supplementary file 1 — Supplementary material 1 (PDF 679 kb) [file 10147_2017_1193_MOESM1_ESM.pdf]

Basic Items Regarding the Guidelines

I. Cancer staging

1. Vulvar Cancer

In 1988, the International Federation of Gynecology and Obstetrics (FIGO) supplemented the existing classifications for clinical stages of vulvar cancer with classifications for surgical staging of vulvar cancer. Vulvar cancer is usually treated with ordinary surgical procedures and is amenable to histopathological evaluation. FIGO surmised that classifications for surgical staging of vulvar cancer based on histopathological evaluation of associated lymph node metastasis, a key prognostic factor, would provide more accurate prognoses.

(1) Classifications of surgical stages of vulvar cancer (FIGO 1988)

Stage 0: Carcinoma in situ (intraepithelial carcinoma)

Stage I: Tumor confined to the vulva and/or perineum - 2cm or less in the greatest dimension, nodes are not palpable.

Stage II: Tumor confined to the vulva and/or perineum – more than 2cm in the greatest dimension, nodes are not palpable.

Stage III: Tumor of any size with:

1) Adjacent spread to the lower urethra and/or vagina or anus, and/or
2) Unilateral regional lymph-node metastasis

Stage IVa: The tumor invades any of the following:

Upper urethra, bladder mucosa, rectal mucosa , pelvic bone and/or bilateral

regional node metastasis.

Stage IVb: Any distant metastasis including pelvic lymph-node.

Later, as a result of survival analyses of cases on the basis of these classifications, several defects in these classifications were identified. In 1994 Stage I only was revised, with Stage Ia consisting of a tumor of maximum diameter 2cm or less and with stromal invasion of 1mm or less.

(2) Classifications of surgical stages of vulvar cancer (FIGO 1994)

Stage 0: Carcinoma in situ (intraepithelial carcinoma)

Stage I: Lesions 2cm or less in size confined to vulva or perineum. No nodal metastasis.

Stage Ia: Lesions 2cm or less in size confined to vulva or perineum with stromal invasion no greater than 1.0 mm. No nodal metastasis.

Stage Ib: Lesions 2cm or less in size confined to vulva or perineum with stromal invasion greater than 1.0 mm . No nodal metastasis.

Stage II: Tumor confined to the vulva and/or perineum, more than 2cm in the greatest dimension, with no nodal metastasis.

Stage III: Tumor of any size with:

1) Adjacent spread to the lower urethra and/or vagina or anus, and/or
2) Unilateral regional lymph-node metastasis

Stage IVa: The tumor invades any of the following:

Upper urethra, bladder mucosa, rectal mucosa , pelvic bone and/or bilateral regional node metastasis.

Stage IVb: Any distant metastasis including pelvic lymph-node.

Note: The depth of invasion is defined as the measurement of the tumor from the epithelial–stromal junction of the adjacent most superficial dermal papilla to the deepest point of invasion.

These classifications of staging present the following problems:
(1)The classifications are not reflected in differences of prognosis for each stage. In particular, the difference in survival rate between Stage I and Stage II is slight. (2)Stage III contains two prognostic groups with favorable prognoses and poor prognoses. (3)The status and numbers of lymphatic-node metastases are not reflected in the classifications of staging. [1, 2]
FIGO adopted a new set of classifications for surgical staging to resolve these problems in 2008[3]. Stage I was defined as a tumor localized in the vulva, tumors 2cm in diameter or less were merged with those 2cm or greater in old stage II. Stage IA was left unchanged, while Stage Ib consisted of cases of tumors 2cm in diameter or greater or stromal invasion exceeding 1.0mm. This division was based on data from the Surveillance, Epidemiology and End Results Program (SEER) program in the United States, which reported that even tumors 8cm in diameter presented good prognosis with no lymph-node metastasis[1]. Stage II consisted of progression to adjacent perineal tissues but with no lymph-node metastasis, regardless of tumor diameter, while Stage III was defined as associated lymph-node metastasis regardless of tumor diameter and progression to adjacent perineal tissues. Stage III was divided into subclassification, IIIa, IIIb and IIIc, based on the number and size of metastasized lymph nodes and whether extracapsular invasion presented. Stage IV consisted of progression to the upper urethra and vagina, or accompaniment

by distant metastasis. This stage was divided into IVa, consisting of invasion to the upper urethra, rectum or pelvic bone and metastasis, and/or fixed or ulcerated inguinal lymph nodes; and IVb, consisting of distant metastasis, including pelvic lymph-node metastasis. All of these changes were the result of argument in the oncological community on the problems with the FIGO 1988 staging classifications.

JSOG commissioned a translation of the FIGO 2008 classifications of surgical staging, which is adopted in 2014[4]. The Classifications of surgical staging (JSOG2014, FIGO 2008) [4] are used in the present guideline.

(3) Classifications of surgical advanced stages (JSOG2014, FIGO 2008) [4]

Stage I Tumor confined to the vulva

IA Lesions ≤2 cm in size, confined to the vulva or perineum and with stromal invasion ≤1.0 mm, no nodal metastasis

IB Lesions >2 cm in size or with stromal invasion >1.0 mm, confined to the vulva or perineum, with negative nodes

Stage II Tumor of any size with extension to adjacent perineal structures (1/3 lower urethra, 1/3 lower vagina, anus) with negative nodes

Stage III Tumor of any size with or without extension to adjacent perineal structures (1/3 lower urethra, 1/3 lower vagina, anus) with positive inguino-femoral lymph nodes

IIIA (i) With 1 lymph node metastasis (≥5 mm), or

(ii) 1–2 lymph node metastasis(es) (<5 mm)

IIIB (i) With 2 or more lymph node metastases (≥5 mm), or

(ii) 3 or more lymph node metastases (<5 mm)

IIIC With positive nodes with extracapsular spread

Stage IV Tumor invades other regional (2/3 upper urethra, 2/3 upper vagina), or distant structures

IVA Tumor invades any of the following:

(i) upper urethral and/or vaginal mucosa, bladder mucosa, rectal mucosa, or fixed to pelvic bone, or

(ii) fixed or ulcerated inguino-femoral lymph nodes

IVB Any distant metastasis including pelvic lymph nodes

Note: The depth of invasion is defined as the measurement of the tumor from the epithelial–stromal junction of the adjacent most superficial dermal papilla to the deepest point of invasion.

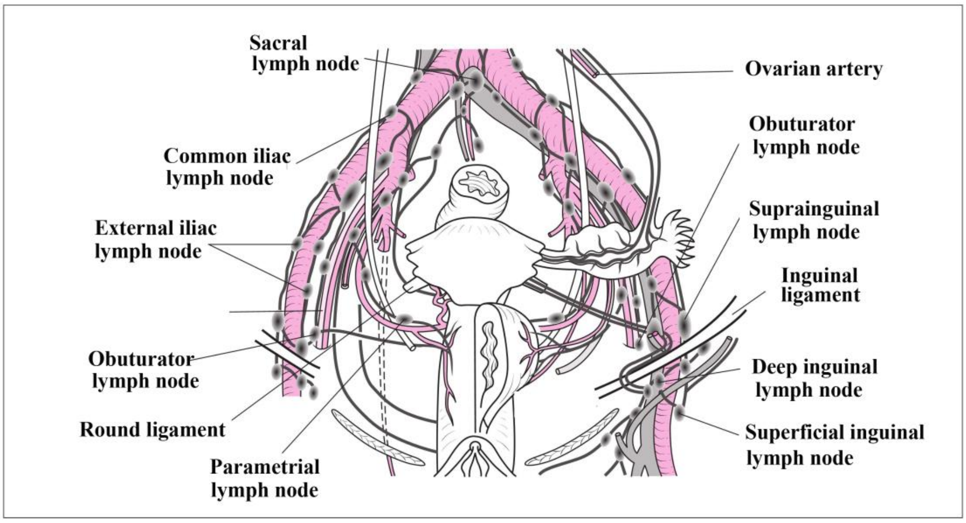

Note:

Regional lymph nodes: Inguinal lymph nodes (superficial inguinal lymph nodes and deep inguinal lymph nodes) (Figure 1)

In the FIGO classifications of staging (2008), regional lymph nodes of vulvar

cancer are listed as the “inguinofemoral nodes” or “inguinal and femoral lymph nodes.” However, the femoral lymph nodes are described as follows: “The femoral nodes are situated medial to the femoral vein within the fossa ovalis”[5], “Deep femoral nodes are located medially along the femoral vessels.” [6], and “Deep femoral nodes, which are by classic teaching located beneath the cribriform fascia…” [7]

The lymph nodes in these locations are defined in the morphology code on lymph nodes of the Japan Society of Clinical Oncology (JSCO) as superficial inguinal lymph nodes and deep inguinal lymph nodes[8]. The Guidelines adopt the JSCO terminology, defining these tissues as superficial inguinal lymph nodes and deep inguinal lymph nodes

(5) TNM Categories, 7th Edition (Union for International Cancer Control (UICC), 2009 [9]

UICC’s TNM categories are based on a classification system applied to all malignant tumors of visceral organs. TNM is a combination of “T,” which refers to primary lesions of tumors and their degree of progression; “N,” which refers to regional lymph nodes; and “M,” which refers to distant metastasis. Because the determination of stage in vulvar cancer is based on surgical findings, the normal approach is to use pathological TNM (pTNM) classification, denoted as pT, pN and pM.

■T: Primary lesions

TX Primary tumor cannot be assessed

T0 No evidence of primary tumor

Tis* Carcinoma in situ (preinvasive carcinoma)

T1a Lesions 2 cm or less in size, confined to the vulva or perineum and with stromal invasion 1.0 mm or less**

T1b Lesions more than 2 cm in size or any size with stromal invasion more than 1.0 mm, confined to the vulva or perineum

T2*** Tumor of any size with extension to adjacent perineal structures (lower/distal 1/3 urethra, lower/distal 1/3 vagina, anal involvement)

T3**** Tumor of any size with extension to any of the following: upper/proximal 2/3 of urethra, upper/proximal 2/3 vagina, bladder mucosa, rectal mucosa, or fixed to pelvic bone

*Note: FIGO no longer includes Stage 0 (Tis).

**Note: The depth of invasion is defined as the measurement of the tumor from the epithelial–stromal junction of the adjacent most superficial dermal papilla to the deepest point of invasion.

***FIGO uses the classification T2/T3. This is defined as T2 in TNM.

****FIGO uses the classification T4. This is defined as T3 in TNM.

■N: Regional lymph nodes (superficial inguinal lymph nodes and deep inguinal lymph nodes).

NX Regional lymph nodes cannot be assessed

N0 No regional lymph node metastasis

N1 One or two regional lymph nodes with the following features

- N1a 1 or 2 lymph node metastases each 5 mm or less
- N1b One lymph node metastasis 5 mm or greater

N2　Regional lymph node metastasis with the following features

N2a　Three or more lymph node metastases each less than 5 mm

N2b　Two or more lymph node metastases 5 mm or greater

N2c　Lymph node metastasis with extra-capsular spread

N3　Fixed or ulcerated regional lymph node metastasis

■M: Distant metastasis

M0　No distant metastasis

M1　Distant metastasis　(including pelvic lymph node metastasis)

(6) Relation of FIGO staging classifications and TNM categories in vulvar cancer.

FIGO classifications of stages	TNM categories (7th Edition, UICC)		
	T	N	M
I	T1	N0	M0
Ia	T1a	N0	M0
Ib	T1b	N0	M0
II	T2	N0	M0
IIIa	T1, T2	N1a, N1b	M0
IIIb	T1, T2	N2a, N2b	M0
IIIc	T1, T2	N2c	M0
IVa	T1, T2	N3	M0
	T3	Any N	M0
IVb	Any T	Any N	M1

2. Vaginal Cancer

It is stipulated that a tumor is diagnosed as cervical cancer if it extends as far as the cervix, and as vulvar cancer if it extends to the vulva[10)]. Because of these definitions, cases diagnosed as vaginal cancer are few.

FIGO adopted the classifications of clinical staging in 1971[11, 12)], but has not revised them since then. Because about 75% of cases of vaginal cancer are diagnosed as Stage II to IV and most of them are treated by radiation therapy, the classifications of clinical staging are adopted for vaginal cancer as they are for cervical cancer. It follows that pelvic examinations, colposcopy, cystoscopy, proctoscopy and X-ray examinations can be used for staging, while image-based examinations such as CT and MRI can only be used to decide treatment plans, the FIGO stages must not be changed by the findings of these diagnostic methods[13)].

JSOG prepared a Japanese translation of the 1971 FIGO classifications of clinical stages and adopted them in 2014[4)] and used in the present guideline.

(1) Classifications of clinical stages (FIGO 1971, JSOG 2014) [4, 11)]

Stage I: The carcinoma is limited to the vaginal wall.

Stage II: The carcinoma has involved subvaginal tissue, but has not extended to the pelvic wall.

Stage III: The carcinoma has extended to the pelvic wall.

Stage IV: The carcinoma has extended beyond the true pelvis, or has involved the mucosa of the bladder or rectum; bullous edema is not sufficient to allow

classification as to stage IV.

Stage IVa: The growth spreads to adjacent organs and/or direct invasion beyond the true pelvis.

Stage IVb: The growth spreads to distant organs.

Regional lymph node in vaginal cancer (Figure 1)

In cases of the primary lesion is in the upper two-thirds of the vagina: Pelvic lymph nodes (suprainguinal lymph nodes, obturator lymph nodes, internal iliac lymph nodes, external iliac lymph nodes, common iliac lymph nodes, sacral lymph nodes)

In cases of the primary lesion is in the lower third of the vagina: inguinal lymph nodes (superficial inguinal lymph nodes, deep inguinal lymph nodes:

(3) TNM categories (*UICC 7th Edition*, 2009) [14)]

Because determination of stages of vaginal cancer is based on pretreatment findings, the clinical TNM categories are used.

■T: Primary lesions

TX: Primary tumor cannot be assessed.

T0: No evidence of primary tumor.

Tis: Carcinoma in situ (preinvasive).

T1: Tumor confined to the vagina.

T2: Tumor invades paravaginal tissue but not to pelvic wall.

T3: Tumor extends to pelvic wall.

T4: The tumor invades mucosa of the bladder or rectum and/or extends beyond

the true pelvis (bullous edema is not sufficient evidence to classify a tumor as T4).

Note: FIGO no longer includes stage 0(Tis)

Note: Pelvic wall was defined as muscle, fascia, neurovascular structures, or skeletal portions of the bony pelvis. On rectal examination, there is no cancer -free space between the tumor and the pelvic wall.

■N—Regional lymph nodes

NX: Regional lymph nodes cannot be assessed.

N0: No regional lymph node metastasis.

N1: Metastasis to the regional lymph nodes.

Regional lymph nodes are as follows:

Upper two-thirds of vagina: Pelvic lymph nodes (suprainguinal lymph nodes, obturator lymph nodes, internal iliac lymph nodes, external iliac lymph nodes, common iliac lymph nodes, sacral lymph nodes)

Lower third of vagina: Inguinal lymph nodes (superficial inguinal lymph nodes, deep inguinal lymph nodes)

■M: Distant metastasis

M0: No distant metastasis.

M1: Distant metastasis.

3. Vulvar malignant melanomas

Malignant melanomas tend to metastasize at an early stage. Their prognosis is poor. Because factors such as depth of cancer invasion, thickness,

presence/absence of ulcers and lymphatic metastasis are all related to the prognosis for a malignant melanoma[15)], malignant melanomas are biologically different from vulvar cancers, typical of which are squamous carcinomas, and a line needs to be drawn between the two diseases. Malignant melanomas usually arise in skin as cutaneous type, and less frequently occur on the epithelium of the lips, intraoral area, eyelids, nasal cavity and vulva, which are called mucosal melanoma and distinct from the cutaneous type. In Japan the Japanese Skin Cancer Society (JSCS) has published its *Guidelines on Handling of Malignant Skin Tumors*[16)], which describes how to handle malignant melanomas.

The TNM categories for malignant melanomas apply to cutaneous malignant melanomas. Because unique categories of staging have not been established for vulvar malignant melanomas and for malignant melanomas originating in the mucous membrane, the TNM categories for cutaneous malignant melanomas are applied to vulvar malignant melanomas, which are classified as mucosal melanoma. The present guideline uses the TNM categories (American Joint Committee on Cancer (AJCC), 2009) [16)]. The UICC TNM categories (2009) are almost identical to the 2002 categories, so the AJCC TNM categories are used. Depth of invasion was measured in reference to the (*Guidelines on Handling of Malignant Skin Tumors, 2nd Edition* (August 2010, Kanehara & Co., Ltd.

The TNM categories (American Joint Committee on Cancer (AJCC), 2009)

■T: Primary lesions (Figure 2)

TX: Primary tumor cannot be assessed (e.g., curettage or severely regressed melanoma)

T0: No evidence of primary tumor.

Tis: Melanoma in situ.

T1: Melanomas 1 mm or less in thickness.

T1a: No ulcers, and mitosis less than $1/mm^2$.

T1b: Ulcers present, or mitosis $1/mm^2$ or more.

T2: Melanomas, 1mm < tumor thickness ≤ 2mm.

T2a: No ulcers.

T2b: Ulcers present.

T3: Melanomas, 2mm < tumor thickness ≤ 4mm.

T3a: No ulcers.

T3b: Ulcers present.

T4: Tumor thickness >4mm.

T4a: No ulcers.

T4b: Ulcers present.

■N: Associated lymph nodes

NX: The regional nodes cannot be assessed..

N0: No metastasis in the regional lymph nodes, satellite metastasis* or in-transit metastasis** is not confirmed.

N1: Metastasis is confirmed in one regional lymph node.

N1a: Microscopic metastasis.

N1b: Gross metastasis.

N2: Metastasis is confirmed in 2–3 regional lymph nodes, or satellite or in-transit metastasis is confirmed that is not accompanied by lymph-node metastasis.

N2a: Two or three microscopic metastases.

N2b: Two or three gross metastases.

N2c: Satellite or in-transit metastasis without lymph-node metastasis.

N3: Four or more metastatic regional lymph nodes, or matted nodes, or satellite or in-transit metastasis accompanied by lymph-node metastasis is confirmed.

Note: Associated lymph nodes are inguinal lymph nodes (superficial inguinal lymph nodes, deep inguinal lymph nodes) and iliac lymph nodes.

* Skin metastasis within 2cm of the primary tumor

** Skin metastasis occurring between the primary tumor and regional lymph nodes except for satellite metastasis.

■M—Distant metastasis

M0: No detectable evidence of distant metastases.

M1: Distant metastasis is confirmed.

M1a: Metastasis to skin, subcutaneous, or distant lymph nodes.

M1b: Metastasis to lung.

M1c: Metastasis to all other visceral sites or distant metastasis to any site combined with an elevated serum LDH.

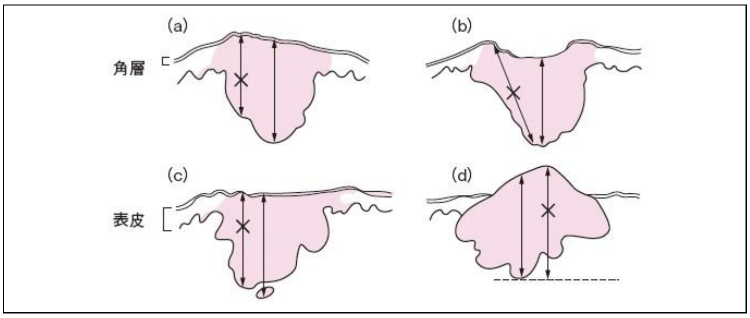

Figure 2 Methods of measuring tumor thickness

A micrometer (disciform glass plate etched with gradations) is placed inside the lens-barrel of an eyepiece mounted on a microscope, and tumor thickness is measured using this microscope. In the direction perpendicular to the epithelium, the distance between the top of the granular layer of the epithelium and the deepest tumor cells (abnormal melanocytes) is measured. Multiple sections of the lesion are measured, focusing on cross-sections where tumor thickness is greatest, and the largest of these sections is taken (a). If the surface is ulcerated, the above starting point is taken to be the surface of an ulcer (b, d). If microsatellites (microscopic satellite lesions) are evident on the bottom of the primary lesion, these are included and the largest section is taken (c). Do not measure diagonally (b) or measure a longer distance using a line extended horizontally (d). (The lines marked with an "**X**" are examples of incorrect measurements of tumor thickness.)

II. Histological Classification

Rather than create own histological classification system for tumors of the vulva and vagina, Japan adopts the WHO classifications. After using the same classification since 2003, WHO published a revised classification in 2014. The Guidelines incorporate both the 2003 WHO classifications (WHO Classification of Tumours of the Vulva 2003, WHO Classification of Tumours of the Vagina 2003) and the 2014 WHO classification (WHO Classification of Tumours of the Vulva 2014, WHO Classification of Tumours of the Vagina 2014).

1. 2003 WHO classifications

(1) WHO Histological Classification of Tumours of the Vulva 2003

Epithelial tumours

Squamous and related tumours and precursors

Squamous cell carcinoma,

not otherwise specified 8070/ 3

Keratinizing 8071/ 3

Non-keratinizing 8072/ 3

Basaloid 8083/ 3

Warty 8051/ 3

Verrucous 8051/ 3

Keratoacanthoma-like

Variant with tumour giant cells

Others

Basal cell carcinoma 8090/ 3

Squamous intraepithelial neoplasia

Vulvar intraepithelial neoplasia

(VIN) 3/ 8077/ 2

Squamous cell carcinoma in situ 8070/ 2

Benign squamous lesions

Condyloma acuminatum

Vestibular papilloma

(micropapillomatosis) 8052/ 0

Fibroepithelial polyp

Seborrheic and inverted follicular keratosis

Keratoacanthoma

Glandular tumours

Paget's disease 8542/ 3

Bartholin gland tumours

Adenocarcinoma 8140/ 3

Squamous cell carcinoma 8070/ 3

Adenoid cystic carcinoma 8200/3

Adenosquamous carcinoma 8560/ 3

Transitional cell carcinoma 8120/ 3

Small cell carcinoma 8041/ 3

Adenoma 8140/ 0

Adenomyoma 8932/ 0

Others

Tumours arising from specialized anogenital mammary-like glands

Adenocarcinoma of mammary gland type 8500/ 3

Papillary hidradenoma 8405/ 0

Others

Adenocarcinoma of Skene gland origin 8140/ 3

Adenocarcinomas of other types

8140/ 3

Adenoma of minor vestibular glands 8140/ 0

Mixed tumour of the vulva 8940/ 0

Tumours of skin appendage origin

Malignant sweat gland tumours 8400/ 3

Sebaceous carcinoma 8410/ 3

Syringoma 8407/ 0

Nodular hidradenoma 8402/ 0

Trichoepithelioma 8100/ 0

Trichilemmoma 8102/ 0

Others

Soft tissue tumours

Sarcoma botryoides 8910/ 3

Leiomyosarcoma 8890/ 3

Proximal epithelioid sarcoma 8804/ 3

Alveolar soft part sarcoma 9581/ 3

Liposarcoma 8850/ 3

Dermatofibrosarcoma protuberans 8832/ 3

Deep angiomyxoma 8841/ 1

Superficial angiomyxoma 8841/ 0

Angiomyofibroblastoma 8826/ 0

Cellular angiofibroma 9160/ 0

Leiomyoma 8890/ 0

Granular cell tumour 9580/ 0

Others

Melanocytic tumours

Malignant melanoma 8720/ 3

Congenital melanocytic naevus 8761/ 0

Acquired melanocytic naevus 8720/ 0

Blue naevus 8780/ 0

Atypical melanocytic naevus of the genital type 8720/ 0

Dysplastic melanocytic naevus 8727/ 0

Miscellaneous tumours

Yolk sac tumour 9071/ 3

Merkel cell tumour 8247/ 3

Peripheral primitive neuroectodermal tumour/ 9364/ 3

Ewing tumour 9260/ 3

Haematopoetic and lymphoid tumours

Malignant lymphoma (specify type)

Leukaemia (specify type)

Secondary tumours

1. Morphology code of the International Classification of Diseases for Oncology (ICD-O) (921) and the Systematized Nomenclature of Medicine (http://snomed.org). Behaviour is coded / 0 for benign tumours, / 2 for in situ carcinomas and grade 3 intraepithelial neoplasia, / 3 for malignant tumours, and / 1 for borderline or uncertain behaviour.
2. Intraepithelial neoplasia does not have a generic code in ICD-O. ICD-O codes are only available for lesions categorized as squamous intraepithelial neoplasia grade 3 (e.g. intraepithelial neoplasia/ VIN grade 3) = 8077/ 2; squamous cell carcinoma in situ 8070/ 2.
(From *WHO Classification of Tumours: Pathology and Genetics of the Tumours of the Breast and Female Genital Organs* (2003, IARC Press))

(2) WHO histological classification of tumours of the vagina 2003

Epithelial tumours

Squamous tumours and precursors

Squamous cell carcinoma,
not otherwise specified 8070/ 3
Keratinizing 8071/ 3
Non-keratinizing 8072/ 3
Basaloid 8083/ 3
Verrucous 8051/ 3
Warty 8051/ 3
Squamous intraepithelial neoplasia
Vaginal intraepithelial neoplasia 3 / 8077/ 2
Squamous cell carcinoma in situ 8070/ 2
Benign squamous lesions
Condyloma acuminatum
Squamous papilloma (vaginal micropapillomatosis) 8052/ 0

Fibroepithelial polyp

Glandular tumours

Clear cell adenocarcinoma 8310/ 3

Endometrioid adenocarcinoma 8380/ 3

Mucinous adenocarcinoma 8480/ 3

Mesonephric adenocarcinoma 9110/ 3

Müllerian papilloma

Adenoma, not otherwise specified 8140/ 0

Tubular 8211/ 0

Tubulovillous 8263/ 0

Villous 8261/ 0

Other epithelial tumours

Adenosquamous carcinoma 8560/ 3

Adenoid cystic carcinoma 8200/ 3

Adenoid basal carcinoma 8098/ 3

Carcinoid 8240/ 3

Small cell carcinoma 8041/ 3

Undifferentiated carcinoma 8020/ 3

Mesenchymal tumours and tumour-like conditions

Sarcoma botryoides 8910/ 3

Leiomyosarcoma 8890/ 3

Endometrioid stromal sarcoma, low grade 8931/ 3

Undifferentiated vaginal sarcoma 8805/ 3

Leiomyoma 8890/ 0

Genital rhabdomyoma 8905/ 0

Deep angiomyxoma 8841/ 1

Postoperative spindle cell nodule

Mixed epithelial and mesenchymal tumours

Carcinosarcoma (malignant müllerian mixed tumour; metaplastic carcinoma) 8980/ 3

Adenosarcoma 8933/ 3

Malignant mixed tumour resembling synovial sarcoma 8940/ 3

Benign mixed tumour 8940/ 0

Melanocytic tumours

Malignant melanoma 8720/ 3

Blue naevus 8780/ 0

Melanocytic naevus 8720/ 0

Miscellaneous tumours

Tumours of germ cell type

Yolk sac tumour 9071/ 3

Dermoid cyst 9084/ 0

Others

Peripheral primitive neuroectodermal tumour/ Ewing tumour 9364/ 3

Ewing tumour 9260/ 3

Adenomatoid tumour 9054/ 0

Lymphoid and haematopoetic tumours

Malignant lymphoma (specify type)

Leukaemia (specify type)

Secondary tumours

1. Morphology code of the International Classification of Diseases for Oncology (ICD-O) (921) and the Systematized Nomenclature of Medicine (http://snomed.org). Behaviour is coded / 0 for benign tumours, / 2 for in situ carcinomas and grade 3 intraepithelial neoplasia, / 3 for malignant tumours, and / 1 for borderline or uncertain behaviour.
2. Intraepithelial neoplasia does not have a generic code in ICD-O. ICD-O codes are only available for lesions categorized as squamous intraepithelial neoplasia grade 3 (e.g. vaginal intraepithelial neoplasia/ VAIN grade 3) = 8077/ 2; squamous cell carcinoma in situ = 8070/ 2.

(From *WHO Classification of Tumours: Pathology and Genetics of the Tumours of the Breast and Female Genital Organs* (2003, IARC Press))

2. 2014 WHO Classification

(1) WHO Classification of tumours of the vulva 2014 a, b

Squamous cell tumours and precursors

Squamous intraepithelial lesions

Low-grade squamous intraepithelial lesion 8077/ 0

High-grade squamous intraepithelial lesion 8077/ 2

Differentiated-type vulvar intraepithelial neoplasia 8071/ 2*
Squamous cell carcinoma 8070/ 3
Keratinizing 8071/ 3
Non-keratinizing 8072/ 3
Basaloid 8083/ 3
Warty 8051/ 3
Verrucous 8051/ 3
Basal cell carcinoma 8090/ 3
Benign squamous lesions
Condyloma acuminatum
Vestibular papilloma 8052/ 0
Seborrheic keratosis
Keratoacanthoma

Glandular tumours

Paget disease 8542/ 3
Tumours arising from Bartholin and other specialized anogenital glands
Bartholin gland carcinomas
Adenocarcinoma 8140/ 3
Squamous cell carcinoma 8070/ 3
Adenosquamous carcinoma 8560/ 3
Adenoid cystic carcinoma 8200/ 3
Transitional cell carcinoma 8120/ 3
Adenocarcinoma of mammary gland type 8500/ 3
Adenocarcinoma of Skene gland origin 8140/ 3
Phyllodes tumour, malignant 9020/ 3
Adenocarcinomas of other types
Adenocarcinoma of sweat gland type 8140/ 3
Adenocarcinoma of intestinal type 8140/ 3
Benign tumours and cysts
Papillary hidradenoma 8405/ 0
Mixed tumour 8940/ 0
Fibroadenoma 9010/ 0
Adenoma 8140/ 0
Adenomyoma 8932/ 0
Bartholin gland cyst
Nodular Bartholin gland hyperplasia
Other vestibular gland cysts
Other cysts
Neuroendocrine tumours
High-grade neuroendocrine carcinoma
Small cell neuroendocrine carcinoma

8041/ 3

Large cell neuroendocrine carcinoma 8013/ 3

Merkel cell tumour 8247/ 3

Neuroectodermal tumours

Ewing sarcoma 9364/ 3

Soft tissue tumours

Benign tumours

Lipoma 8850/ 0

Fibroepithelial stromal polyp

Superficial angiomyxoma 8841/ 0 *

Superficial myofibroblastoma 8825/ 0

Cellular angiofibroma 9160/ 0

Angiomyofibroblastoma 8826/ 0

Aggressive angiomyxoma 8841/ 0 *

Leiomyoma 8890/ 0

Granular cell tumour 9580/ 0

Other benign tumours

Malignant tumours

Rhabdomyosarcoma

Embryonal 8910/ 3

Alveolar 8920/ 3

Leiomyosarcoma 8890/ 3

Epithelioid sarcoma 8804/ 3

Alveolar soft part sarcoma 9581/ 3

Other sarcomas

Liposarcoma 8850/ 3

Malignant peripheral nerve sheath tumour 9540/ 3

Kaposi sarcoma 9140/ 3

Fibrosarcoma 8810/ 3

Dermatofibrosarcoma protuberans 8832/ 1 *

Melanocytic tumours

Melanocytic naevi

Congenital melanocytic naevus 8761/ 0

Acquired melanocytic naevus 8720/ 0

Blue naevus 8780/ 0

Atypical melanocytic naevus of genital type 8720/ 0

Dysplastic melanocytic naevus 8727/ 0

Malignant melanoma 8720/ 3

Germ cell tumours

Yolk sac tumour 9071/ 3

Lymphoid and myeloid tumours

Lymphomas

Myeloid neoplasias **Secondary tumours**

a The morphology codes are from the International Classification of Diseases for Oncology (ICD-O) (575A). Behaviour is coded / 0 for benign tumours, / 1 for unspecified, borderline or uncertain behaviour, / 2 for carcinoma in situ and grade III intraepithelial neoplasia and / 3 for malignant tumours; b The classification is modified from the previous WHO classification of tumours (1906A), taking into account changes in our understanding of these lesions; These new codes were approved by the IARC/ WHO Committee for ICD-O in 2013.
(From *WHO Classification of Tumours of Female Reproductive Organs, Fourth Edition* (2014, IARC Press))

(2) WHO Classification of tumours of the vagina 2014 a, b

Epithelial tumours

Squamous cell tumours and precursors

Squamous intraepithelial lesions

Low-grade squamous intraepithelial lesion 8077/ 0

High-grade squamous intraepithelial lesion 8077/ 2

Squamous cell carcinoma, NOS 8070/ 3

Keratinizing 8071/ 3

Non-keratinizing 8072/ 3

Papillary 8052/ 3

Basaloid 8083/ 3

Warty 8051/ 3

Verrucous 8051/ 3

Benign squamous lesions

Condyloma acuminatum

Squamous papilloma 8052/ 0

Fibroepithelial polyp

Tubulosquamous polyp 8560/ 0

Transitional cell metaplasia

Glandular tumours

Adenocarcinomas

Endometrioid carcinoma 8380/ 3

Clear cell carcinoma 8310/ 3

Mucinous carcinoma 8480/ 3

Mesonephric carcinoma 9110/ 3

Benign glandular lesions

Tubovillous adenoma 8263/ 0

Villous adenoma 8261/ 0

Müllerian papilloma

Adenosis

Endometriosis

Endocervicosis

Cysts

Other epithelial tumours

Mixed tumour 8940/ 0

Adenosquamous carcinoma 8560/ 3

Adenoid basal carcinoma 8098/ 3

High-grade neuroendocrine carcinoma

Small cell neuroendocrine carcinoma 8041/ 3

Large cell neuroendocrine carcinoma 8013/ 3

Mesenchymal tumours

Leiomyoma 8890/ 0

Rhabdomyoma 8905/ 0

**Leiomyosarcoma**	8890/ 3
**Rhabdomyosarcoma, NOS**	8900/ 3
Enbryonal rhabdomyosarcoma	8910/ 3
**Undifferentiated sarcoma**	8805/ 3
**Angiomyofibroblastoma**	8826/ 0
**Aggressive angiomyxoma**	8841/ 0
**Myofibroblastoma**	8825/ 0
**Tumour-like lesions**	
Postoperative spindle cell nodule	
**Mixed epithelial and mesenchymal tumours**	
**Adenosarcoma**	8933/ 3
**Carcinosarcoma**	8980/ 3
**Lymphoid and myeloid tumours**	
**Lymphomas**	
Myeloid neoplasias	
**Melanocytic tumours**	
**Naevi**	
Melanocytic naevus	8720/ 0
Blue naevus	8780/ 0
**Malignant melanoma**	8720/ 3
**Miscellaneous tumours**	
**Germ cell tumours**	
Mature teratoma	9084/ 0
Yolk sac tumour	9071/ 3
**Others**	
Ewing sarcoma	9364/ 3
Paraganglioma	8693/ 1
**Secondary tumours**	

a. The morphology codes are from the International Classification of Diseases for Oncology (ICD-O) (575A). Behaviour is coded / 0 for benign tumours, / 1 for unspecified, borderline or uncertain behaviour, / 2 for carcinoma in situ and grade III intraepithelial neoplasia and / 3 for malignant tumours; b The classification is modified from the previous WHO classification of tumours (1906A), taking into account changes in our understanding of these lesions; * These new codes were approved by the IARC/ WHO Committee for ICD-O in 2013.

From *WHO Classification of Tumours of Female Reproductive Organs, Fourth*

Edition (2014, IARC Press))

Handling of Intraepithelial Neoplasias in the WHO Classifications of 2003 and 2014

In the WHO histological classification of 2014, clear categories are provided for intraepithelial neoplasias, but the nomenclatures of the categories are changed. In particular, WHO 2014 distinguishes between vulvar intraepithelial neoplasias that are related to the human papillomavirus (HPV) and those that are not.

(1) Vulva

WHO 2003	WHO 2014
Non-HPV-related	
Simplex (differentiated) vulvar intraepithelial neoplasia (VIN)	Differentiated VIN
HPV-related	
VIN 1	Low-grade squamous intraepithelial lesion (LSIL)
VIN 2	High-grade squamous intraepithelial lesion (HSIL)
VIN 3	

VIN: Vulvar intraepithelial neoplasia

LSIL: Low-grade squamous intraepithelial lesion

HSIL: High-grade squamous intraepithelial lesion

(2) Vagina

WHO 2003	WHO 2014

Vaginal intraepithelial neoplasia (VAIN) 1	LSIL
VAIN 2	HSIL
VAIN 3 HSIL	

VAIN: Vaginal intraepithelial neoplasia

LSIL: Low-grade squamous intraepithelial lesion

HSIL: High-grade squamous intraepithelial lesion

III Surgical Therapy

1. Vulvar tumors (CQ01, CQ02, CQ03, CQ04, CQ05, CQ06, CQ15)

1) Laser vaporization

In this surgical technique, sufficient margin is established around the focus of disease, then the lesion and surrounding skin are vaporized using laser.

2) Wide local excision

Obtaining sufficient margin around the tumor, the epithelium, mucous membrane and dermis are excised.

3) Simple vulvectomy (Figure 3)

This routine surgery excises the entire vulva to excise all foci of disease. Unless invasive carcinoma is suspected, there is no need to excise deep hypodermal layers.

4) Radical vulvar local excision (Figure 4)

Obtaining an excision margin of about 2cm in the normal skin and vaginal wall, the hypodermis is excised as far as the fascia, to a similar depth as for a radical vulvectomy. In the case of invasive carcinoma, modified radical vulvectomy, in which a part of normal area was left intact like as radical hemivulvectomy, is included in this technique. To conduct an inguinal lymphadenectomy, it is necessary to operate on the inguinal skin separately.

5) Radical vulvectomy and inguinal lymphadenectomy (Figures 5a and 5b)

Obtaining a wide excision margin around the tumor on the inside and outside, a ring-shaped incision is made around the vulva following the outside of the labia major. The hypodermis is cut as far as the superficial fascia of the perineum (Colles fascia) and the adipose tissue is removed from the fascia and excised in

the direction of the vagina. On the inside, an incision is made around the upper external urethral orifice, cutting along the entrance to the vagina. The methods of skin incision used in an inguinal lymphadenectomy are classified as follows.

1) Separate incision (Figure 5a)

Separate incision uses the triple-incision approach, in which incision lines are entered on both sides of the groin, independently from the incision line used to excise the vulva.

If lymph-node metastasis is suspected as a result of palpation of the groin, an incision is made to excise the skin directly over the metastasized lymph nodes. If lymph-node metastasis is not suspected, excision of the skin is avoided.

2) En bloc incision (Figure 5b)

An incision to excise the vulva is extended to both sides of the groin and a longhorn-shaped excision is made from both sides of the anterior superior iliac spine along the inguinal ligament to the mons veneris. The hypodermis is completely excised from the vulva to the adipose tissue, including the lymphatic tissue, of the groin. If lymph-node metastasis is suspected, the skin directly above is excised in the same way as for a separate incision.

6) Pelvic exenteration

In advanced cases, the cancer may infiltrate the peripheral internal organs in the pelvis. In such cases extirpative surgery is undertaken on the vagina, uterus, urinary bladder, rectum and anus.

7) Reconstructive surgery

When a patient's vulva is lost due to radical vulvectomy, cerclage, epidermization and skin grafting are conducted.

8) Inguinal lymphadenectomy

The fascia lata in the femoral triangle are called the cribriform fascia. The inguinal lymph nodes that are superficial to the cribriform fascia are called the superficial inguinal lymph nodes, while those that are deeper are the deep inguinal lymph nodes. The lymph node close to the most rostral inguinal ligament inside the femoral vein is the Cloquet node (also called the Rosenmüller node). Normally all of these lymph nodes are removed.

9) Pelvic lymphadenectomy

Surgery of vulvar cancer generally involves extending the incision line of the inguinal lymphadenectomy to remove the pelvic lymph nodes via the retroperitoneum.

10) Sentinel-lymph-node biopsy

The sentinel lymph nodes are so called because they are the first place malignant cells arrive at when they pass through the lymph ducts. If metastasis has not reached these lymph nodes, removal of other lymph nodes can be omitted.

11) Mapping biopsy [16)]

A mapping biopsy is conducted to estimate the boundaries of a focus of disease whose boundaries are unknown, such as vulvar Paget's disease. In this form of biopsy, biopsies are taken in eight directions in a radial pattern around the focus of disease, or a uniform part 1–3cm in a preset direction is biopsied.

In the Guidelines, the terminology used regarding excisions is defined as follows.

・Excision margin (Figure 6: a)

The excision margin is the distance from the margin (edge) of the tumor to

the area of surgical excision/incision.

・Surgical excision stump (Figure 6: b)

A surgical excision stump is the stump of the tissue that remains after surgical excision.

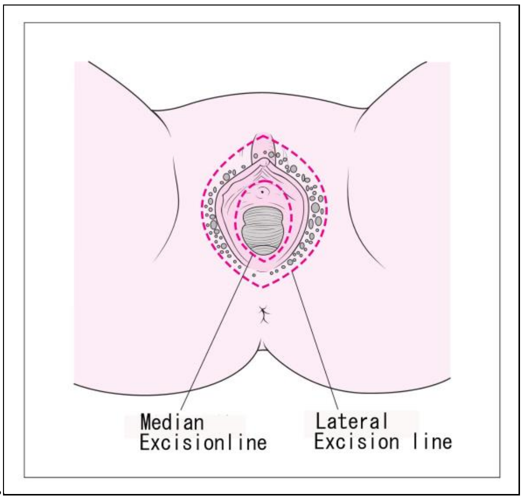

Figure 3　Incision lines in a simple vulvectomy

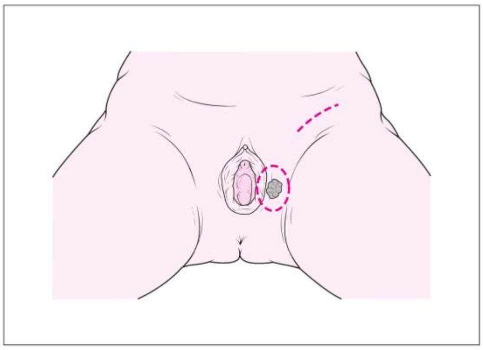

Figure 4　Incision lines in a radical local excision and an inguinal lymphadenectomy

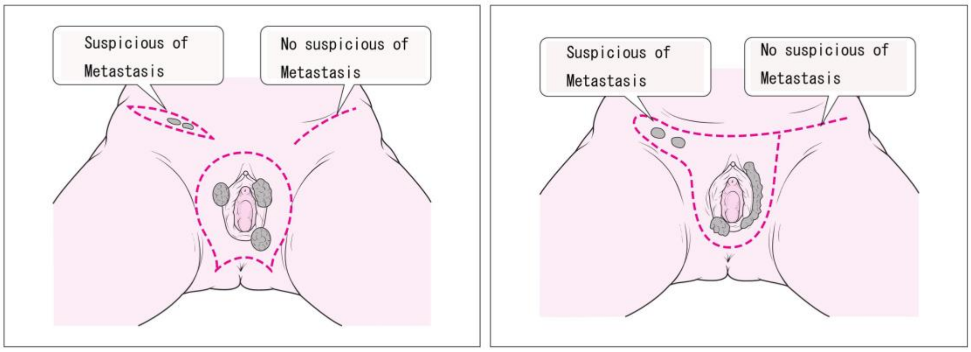

a: Separate incision b: En bloc incision

Figure 5 Incision lines in a radical vulvectomy

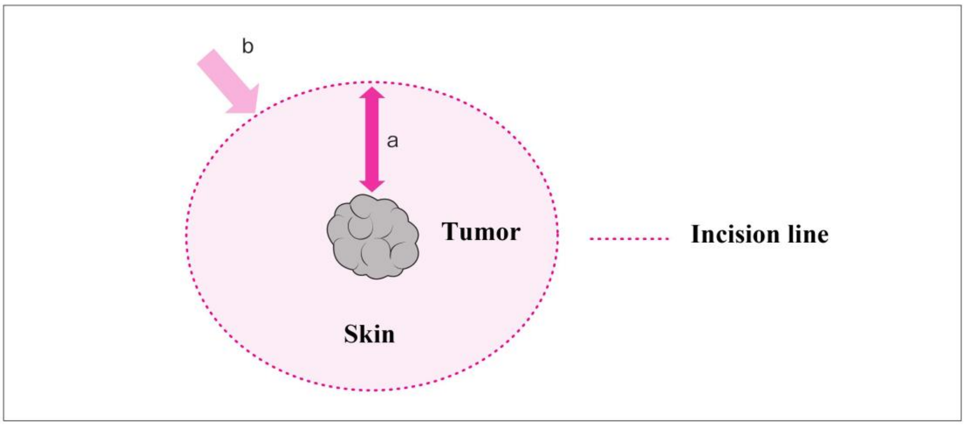

Figure 6 Excision margin (a) and surgical excision stump (b)

2. Vaginal tumors (CQ11, CQ13)

1) Laser vaporization (laser vaporization)

This technique is used to vaporize the skin superficial to the vagina.

2) Partial vaginectomy

Securing a sufficient excision margin around the tumor, the vaginal wall is partially excised.

3) Total vaginectomy

The entire vaginal wall is excised.

4) Radical or modified hysterectomy + vaginectomy + pelvic lymphadenectomy

The vaginal wall is excised along with the uterus, and a pelvic lymphadenectomy is carried out.

5) Pelvic exenteration

This is a composite excision of the vagina, urinary bladder and rectum.

IV Radiation Therapy (CQ07, CQ10, CQ12, CQ15, CQ16)

1. Types of Radiation Therapy

1) Definitive radiation therapy (also called curative radiation therapy)

Radiation therapy is conducted with the aim of curing the patient.

2) Concurrent chemoradiotherapy (CCRT)

Radiation therapy and chemotherapy are performed concurrently.

3) Preoperative irradiation

Irradiation is performed before surgery for locally advanced cancer, to improve the resection rate and minimize invasive surgery, thereby preserving the function of adjacent organs.

4) Postoperative irradiation

When the risk of local recurrence is judged to be high, irradiation is carried out after curative surgery to prevent recurrence.

5) Palliative radiation therapy

Radiation therapy for relief from cancer symptoms.

2. Methods of Radiation Therapy

1) External-beam irradiation

A high-energy beam of radiation is applied from outside the body.

a) Three-dimensional conformal radiation therapy (3D-CRT)

An external-beam irradiation method based on a three-dimensional radiation treatment planning using computed-tomography (CT) image data.

b) Intensity-modulated radiation therapy (IMRT)

An external-beam irradiation method to enable conformal dose distribution to the target, using intensity-modulated radiation beams from multiple directions, based on an inverse treatment planning.

2) Brachytherapy

A small sealed radioactive source is placed near the cancer focus.

1) Intracavitary irradiation

An applicator is inserted into the uterus or vagina and radiation is conducted from within the cavity.

2) Interstitial irradiation

An applicator is inserted into the tumor and adjacent tissues and irradiation is conducted from within the tissues.

3) Image-guided brachytherapy (IGBT)

Brachytherapy based on a 3D radiation treatment planning using image data of CT or magnetic resonance imaging (MRI)

V. Chemotherapy

Currently evidence regarding the use of chemotherapy in treating vulvar cancer, vaginal cancer and similar cancers is scant. As a result, no standard treatment currently exists.

References

1) Hacker, N.F.: Revised FIGO staging for carcinoma of the vulva. *Int J Gynaecol Obstet* 2009; 105: 105─106 (Level IV)

2) Saito, T.: Latest research trends in gynecological cancer: New FIGO staging classification of vulvar cancer in, *Nippon Rinsho* (in Japanese) 2012; 70: 677─682 (Level IV)

3) Pecorelli, S.: Revised FIGO staging for carcinoma of the vulva, cervix, and endometrium. *Int J Gynaecol Obstet* 2009; 105: 103–104 (code)

4) Konishi, I., Aoki, D. Revisions of classifications of surgical staging of ovarian, fallopian-tube and peritoneal cancer and adoption of classifications of surgical staging of vulvar and vaginal cancer, uterine sarcomas and adenosarcomas, *Journal of the JSOG* 2014; 66: 2736─2741 (Code; in Japanese)

5) Hacker, N.F., Eifel, P.J., van der Velden, J., Cancer of the vulva. *Int J Gynaecol Obstet* 2012; 119 (Suppl 2): S90─96 (Level IV)

6) Holschneider, C.H., Berek, J.S., Vulvar Cancer. In: *Berek & Novak's Gynecology 14th ed.* Lippincott Williams & Wilkins, Philadelphia, 2007. pp1549─1580 (Level III)

7) Schilder J.M., Stehman F.B., Invasive Cancer of the vulva. In: DiSaia PJ, Creasman WT, eds. *Clinical Gynecologic Oncology 8th ed. Elsevier* Saunders, Philadelphia, 2012. pp219─244 (Level IV)

8) Proceedings of JSCO, *JSCO Lymph-node Codes*. Kanehara, Tokyo, 2002 (code)

9) Sobin L., Gospodarowicz M., Wittekind C., *TNM Classification of Malignant Tumors, 7th ed.* Wiley-Blackwell, Hoboken, 2010. pp197─201 (code)

10) Hacker, N.F., Eifel, P.J., van der Velden, J., Cancer of the vagina. *Int J Gynaecol Obstet* 2012; 119 (Suppl 2): S97─99 (Level IV)

11) FIGO Committee on Gynecologic Oncology, Current FIGO staging for cancer of the vagina, fallopian tube, ovary, and gestational trophoblastic neoplasia. *Int J Gynaecol Obstet* 2009; 105: 3─4 (code)

12) Iwasaka, T., Malignant tumors of the vulva and vagina. In: Kudo, T., *New Compendium of Women's Medicine,* 38. Ed.: Taketani, Y. et al. Nakayama Shoten, Tokyo, 1998 (Level IV; in Japanese)

13) Bidus, M.A., Elkas J.C., Cervical and Vaginal Cancer. In: *Berek & Novak's Gynecology. 14th ed.* Lippincott Williams & Wilkins, Philadelphia, 2007. pp1444─1456 (Level III)

14) Sobin, L., Gospodarowicz, M., Wittekind, C., *TNM Classification of Malignant Tumors, 7th ed.* Wiley-Blackwell, Hoboken, 2010. pp202─205 (code)

15) Tasseron, E.W., van der Esch, E.P., Hart, A.A., Brutel de la Rivière, G., Aartsen, E.J. A clinicopathological study of 30 melanomas of the vulva. *Gynecol Oncol* 1992; 46: 170─175 (Level III)

16) Proceedings of JSCS, *Code on the Handling of Malignant Skin Tumors, 2nd ed.* Kanehara, Tokyo, 2010 (code; in Japanese)
